# The Effect of Cue Frequency, Modality and Rhythmicity on Finger Tapping Behaviour and Movement‐Related Cortical Activity

**DOI:** 10.1111/ejn.70112

**Published:** 2025-04-19

**Authors:** Janne J. A. Heijs, Silvana Huertas‐Penen, Marc M. van Wanrooij, Bettina C. Schwab, Richard J. A. van Wezel, Tjitske Heida

**Affiliations:** ^1^ TechMed Centre, Biomedical Signals and Systems Group University of Twente Enschede the Netherlands; ^2^ Section Neurobiology, Donders Centre for Neuroscience Radboud University Nijmegen the Netherlands; ^3^ Department of Neurophysiology and Pathophysiology University Medical Center Hamburg‐Eppendorf Hamburg Germany; ^4^ OnePlanet Research Center Radboud University Nijmegen the Netherlands

**Keywords:** beta rhythm, cortical activity, cues, electroencephalography, sensorimotor synchronization

## Abstract

Sensorimotor synchronization (SMS) involves the coordination of movements with rhythmic sensory cues. While cue characteristics influence SMS behaviour and neural pathways, their impact on cortical activity beyond motor areas is less understood. This exploratory EEG study examined how various cue characteristics, including cue frequency, modality and rhythmicity, influence behaviour and movement‐related cortical activity in (non‐)motor areas during SMS. Seventeen healthy participants performed finger tapping with cues varying in frequency (slow: 1 Hz, fast: 3.2 Hz), modality (visual, auditory) and rhythmicity (isorhythmic, polyrhythmic). SMS behaviour and movement‐related beta power were evaluated. Key findings include the following: (1) Increasing cue frequency, and therefore movement speed, reduced tap accuracy, especially with visual cues. Slow cues induced strong beta suppression followed by beta rebound after the tap in the contralateral sensorimotor cortex, while fast cues induced a weaker but sustained beta suppression. (2) Auditory cues enabled more accurate tap behaviour and induced stronger beta suppression in the contralateral premotor cortex compared to visual cues. (3) Polyrhythmic auditory cues delayed taps compared to isorhythmic cues, although tap accuracy was similar. Isorhythmic cues enhanced frontoparietal beta power, whereas polyrhythmic cues showed widespread right‐hemispheric beta suppression. Findings suggest discrete and continuous movement processing with slow and fast cues, respectively. Auditory cues offer more sensory guidance, especially at higher frequencies. Endogenous, top‐down control with isorhythmic cues may switch towards stimulus‐driven, bottom‐up control with auditory polyrhythmic cues. Overall, our findings highlight how cue characteristics shape motor behaviour and neural processes, suggesting distinct movement control strategies depending on frequency, modality and rhythmicity.

## Graphical abstract

This EEG study shows how various cue characteristics, including cue frequency, modality and rhythmicity, influence finger tapping behaviour and movement‐related cortical activity in motor, frontal and parietal areas during sensorimotor synchronization. Our findings suggest distinct strategies for movement processing and control depending on cue frequency, sensory modality and rhythmicity.

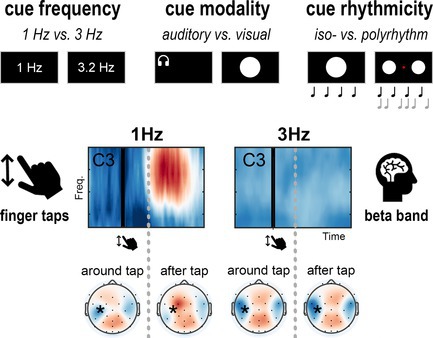

**Abbreviations**
1Hz: refers to conditions with cues presented at 1 Hz
3Hz: refers to conditions with cues presented at 3.2 Hz
AUD: refers to conditions with auditory cues
C: central electrodes (e.g., C3)
CP: centroparietal electrodes (e.g., CP5)
dB: decibel
EEG: electroencephalography
F: frontal electrodes (e.g., F4)
FC: frontocentral electrodes (e.g., FC6)
fMRI: functional magnetic resonance imaging
IQR: interquartile range
ISO: refers to conditions with isorhythmic cues
ISI: inter–stimulus interval
ITI: inter–tap interval
ITI‐CV: coefficient of variation of the inter–tap interval
ITI‐D: discrepancy of the inter–tap interval
MEG: magnetoencephalography
NMA: negative mean asynchrony
P: parietal electrodes (e.g., P4)
PET: positron emission tomography
PO: parieto‐occipital electrodes (e.g., POz)
POLY: refers to conditions with polyrhythmic cues
R: resultant vector length
SD: standard deviation
SMS: sensorimotor synchronization
VIS: refers to conditions with visual cues

## 1 Introduction

We naturally synchronize our movements with the rhythms around us, whether while listening to music, or dancing. This phenomenon, known as sensorimotor synchronization (SMS), requires sensory processing of the external rhythm, extraction of relevant metric structures, integration of this information into the motor program and error correction to accurately synchronize movements to the rhythm (Konoike and Nakamura 2020). SMS is often studied using finger tapping tasks, where participants align the timing of their taps to an external sensory rhythm (Repp 2005), referred to as cues. These studies provide valuable insights into the (patho)physiological mechanisms of motor control and rhythm processing and demonstrate that both tapping behaviour and the neural pathways involved in SMS are influenced by the characteristics of the cue (reviewed in Repp 2005; Repp and Su 2013; Witt et al. 2008). Additionally, electroencephalography (EEG) and magnetoencephalography (MEG) studies have shown that tapping with various metronome frequencies modulates the cortical beta activity within the motor cortices (Alegre et al. 2003; Boonstra et al. 2006; Stegemöller et al. 2016, 2017; Toma et al. 2002). However, less is known about how other cue characteristics, such as sensory modality or rhythmicity, influence movement‐related cortical activity. Moreover, most EEG studies on SMS have primarily focused on motor regions, leaving potential effects in non‐motor areas largely underexplored. Therefore, this EEG study aims to investigate how various cue characteristics, including frequency, sensory modality and rhythmicity, influence tap behaviour and movement‐related cortical activity during SMS, both within and beyond the motor cortices.

Finger tapping behaviour is influenced by the characteristics of the cue provided (reviewed in Repp 2005; Repp and Su 2013). For example, auditory cues typically enable synchronization to higher cue frequencies and result in more accurate tapping compared to visually guided tasks (Jäncke et al. 2000; Jantzen et al. 2005; Kolers and Brewster 1985; Pollok et al. 2009; Whitton and Jiang 2023). In tapping tasks involving complex polyrhythms—consisting of two rhythms with a deviant frequency and/or pitch—participants prefer to synchronize with the rhythm that has the highest frequency or lowest pitch (Handel and Oshinsky 1981; Repp 2003a). Additionally, the brain regions involved in SMS respond differently to various cue characteristics, as demonstrated by a meta‐analysis of functional magnetic resonance imaging (fMRI) and positron emission tomography (PET) studies on finger tapping tasks. While activations in the sensorimotor cortex and supplementary motor area were consistent across tapping tasks, neural activity in other regions, including the premotor, frontal and parietal cortices, varied based on the cue frequency, cue modality and the complexity of the motor task (i.e., unimanual with single or multi‐finger sequences, or bimanual tapping tasks) (Witt et al. 2008). Remarkably, variations in cue characteristics appeared to have a greater influence on the observed differences in functional brain activity than the complexity of the tapping task itself (Witt et al. 2008). This indicates that understanding how different cue characteristics interact with motor control mechanisms is crucial for a comprehensive view of SMS.

Unlike fMRI, which offers higher spatial localization, EEG provides insights into the temporal cortical activity through time‐frequency analysis. During finger tapping, the contralateral sensorimotor cortex reveals a characteristic pattern of movement‐related activity in the beta band (12–30 Hz), important for sensorimotor processing (Kilavik et al. 2013; Neuper and Pfurtscheller 2001; Neuper et al. 2006; Pfurtscheller 2001; Pfurtscheller and Lopes da Silva 1999). Typically, beta power decreases just before and during movement, reflecting activation of the sensorimotor network for movement preparation and execution. After movement, beta power strongly increases above baseline levels, referred to as beta rebound. This beta rebound may indicate either active inhibition or recalibration of motor cortices in preparation for the next movement (Kilavik et al. 2013; Neuper and Pfurtscheller 2001), suggesting that finger taps are prepared and executed as individual, discrete movements (Toma et al. 2002). Interestingly, while a clear beta rebound has been observed during finger tapping with relatively slow auditory cues (below 2 Hz), this rebound is absent with faster cues (above 2 Hz), where beta power remains suppressed below baseline levels (Boonstra et al. 2006; Stegemöller et al. 2016; Toma et al. 2002). This absence of beta rebound with faster movements suggests that the brain processes finger taps continuously as a sequence of movements, rather than timing each tap individually. These previous EEG studies proposed a cut‐off around 2 Hz switching from discrete to continuous motor control as cue frequency, and consequently movement speed, increases (Boonstra et al. 2006; Stegemöller et al. 2016; Toma et al. 2002). Thus, while fMRI and PET studies showed consistent activations in the motor cortex across various tapping tasks (Witt et al. 2008), EEG studies revealed distinct beta band dynamics of the motor cortex in response to cue frequency (Boonstra et al. 2006; Stegemöller et al. 2016; Toma et al. 2002), though the impact of other cue characteristics has been studied less extensively.

Additionally, existing EEG and MEG studies on finger tapping tasks have mainly focused on the movement‐related cortical activity of the motor cortices, with less attention given to other brain regions despite their role in SMS and their sensitivity to variations in cue characteristics (Pollok et al. 2005; Pollok et al. 2006; Witt et al. 2008). For example, the parietal cortex is essential for sensorimotor integration, anticipatory control of movement and feedback processing of errors (Krause et al. 2014; Pollok, Gross, et al. 2008; Pollok et al. 2005). Moreover, enhanced beta power in the frontal cortex has been associated with top‐down control of behaviour during more complex tasks, such as fast compared to slow hand movements (Buschman and Miller 2007; Seeber et al. 2016). Understanding the influence of cue characteristics on beta oscillations in these non‐motor cortices could provide further insights into the mechanisms behind SMS and how they respond to various cue characteristics.

Despite extensive research on the beta activity during finger tapping, we aim to contribute to the SMS literature by addressing two gaps: (1) the limited focus on the influence of other cue characteristics than cue frequency on cortical beta band activity and (2) the evaluation of beta activity in brain regions beyond the commonly studied motor cortices. Therefore, this exploratory EEG study aims to investigate how various cue characteristics, including frequency, sensory modality and rhythmicity, influence tap behaviour and movement‐related cortical beta activity during SMS, both within and beyond motor cortices. Healthy participants performed a unimanual finger tapping task guided by external sensory cues. We examined finger tapping behaviour and the topographical distribution of the beta power across the scalp over time, for eight different cue conditions in a 2 × 2 × 2 design. Each condition was characterized by a unique combination of cue frequency (1 vs. 3.2 Hz), cue modality (auditory vs. visual) and cue rhythmicity (isorhythmic vs. polyrhythmic). We expect that finger tapping guided by more challenging cue conditions (3.2 Hz, visual cues, or polyrhythmic cues) results in a modulation of beta band activity in brain regions related to motor planning and execution (motor cortices), cognitive load (frontal cortex) and sensorimotor integration and error correction (parietal cortex).

## 2 Materials and Methods

### 2.1 Participants

Seventeen right‐handed healthy participants (7 male and 10 female), with an average age of 23.3 ± 2.6 years (mean ± SD), engaged in the study. Exclusion criteria were: a skin disease affecting the skin on the head; epilepsy; a brain surgery in the past; a psychiatric or neurological disease in the past year; the use of drugs affecting the central nervous system (e.g., antidepressants, antipsychotics); or substance abuse in the past year. The data collection of the current study was combined with one of the experimental sessions of another EEG study, using the same EEG equipment (Heijs et al. 2021). Therefore, 13 out of 17 participants performed another experimental protocol of approximately 40 min before the start of the current study (Heijs et al. 2021). Although three additional left‐handed participants performed both experiments, their data were excluded from this study due to the significant hemispheric lateralization of motor control mechanisms (Mutha et al. 2012). Thus, only data from right‐handed participants (*N* = 17) were included in this study.

All participants signed written informed consent before the start of the experiments. The experiments were approved by the Research Ethics Committee of the Faculty of Social Sciences of Radboud University, Nijmegen (ECSW2016‐2208‐413).

### 2.2 Experimental Protocol

**FIGURE 1:**
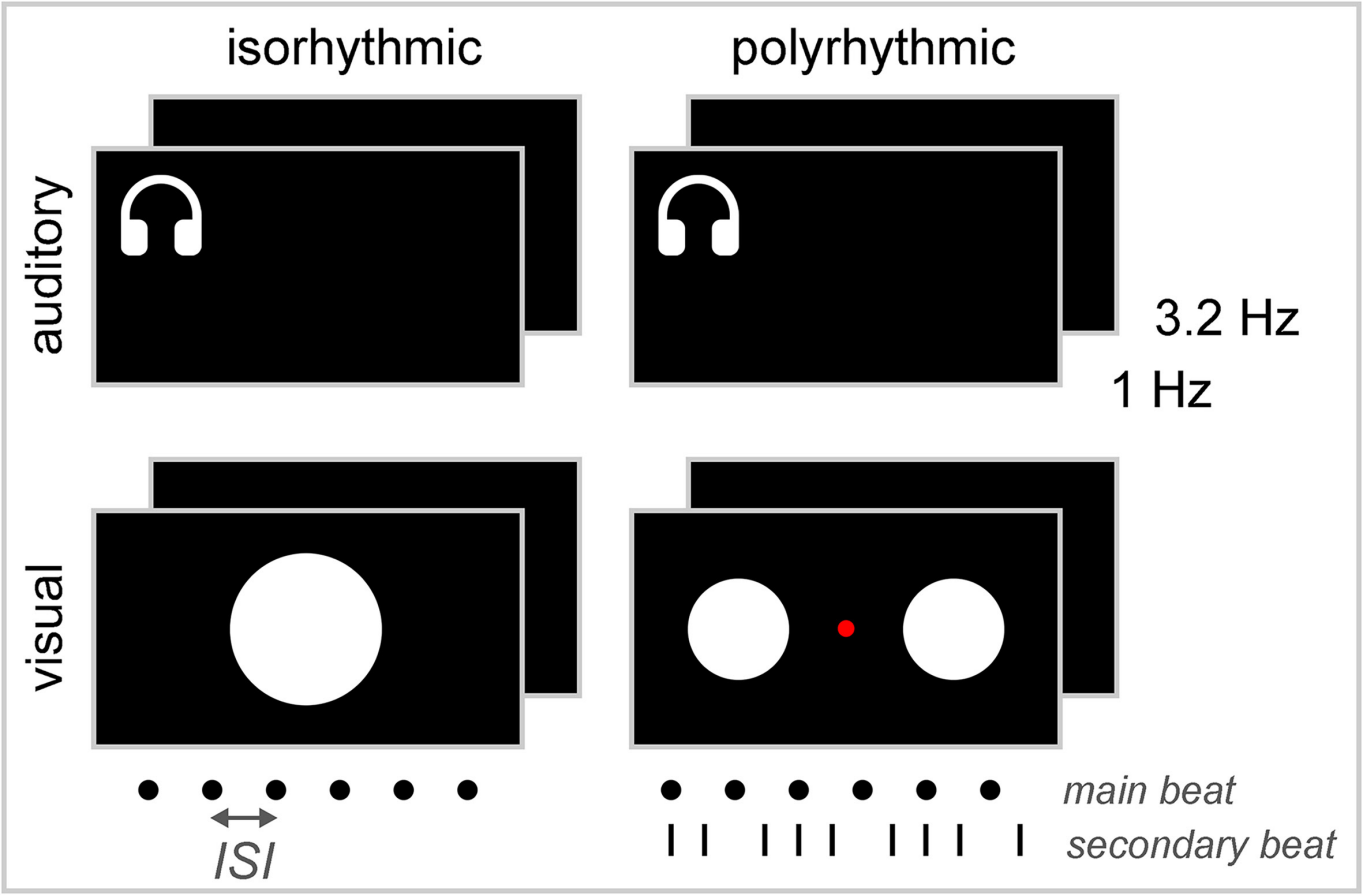
Experimental conditions in a 2 × 2 × 2 design to evaluate the effect of cue frequency (1‐ vs. 3.2‐Hz cues; inter–stimulus interval [ISI] of 1 and 0.3125 s, respectively), cue modality (auditory vs. visual cues) and cue rhythmicity (isorhythmic vs. polyrhythmic cues). Auditory cues consisted of a repetitive tone (pitch A5, i.e., 880 Hz). Visual cues consisted of a flashing white circle, presented at the centre of a black background screen. Isorhythmic cue conditions involved a single rhythm, while a secondary rhythm was added in polyrhythmic cue conditions, with a [2:3] frequency relationship between the main and secondary beats. For visual‐polyrhythmic cues, the left and right circles represent the main and secondary beats, respectively. For auditory‐polyrhythmic cues, the main tone (pitch A5, i.e., 880 Hz) was accompanied by a secondary tone (pitch D3, i.e., 146 Hz).

The experiments were performed in a quiet room with dimmed lights. Participants were asked to take a seat behind a standard laptop (distance to screen approximately 65 cm) with their dominant hand resting on the table while fixating their eyes on the black screen during the experiment. They were instructed to tap with the index finger of their dominant hand following the rhythm of an external, sensory cue. The auditory cues were presented via insert earphones (Etymotic ER1, Etymotic Research Inc., Elk Grove Village, IL, USA), and the visual cues were presented on the laptop's screen (14 in., 60‐Hz refresh rate). A short instruction video was shown to introduce the different cue conditions. Eight different cue conditions were tested in a 2 × 2 × 2 design (Figure 1), each condition characterized by a unique combination of cue frequency, cue modality and cue rhythmicity.

The effect of cue frequency was evaluated by presenting cues at either 1 Hz (i.e., inter–stimulus interval [ISI] of 1 s between consecutive cues) or 3.2 Hz (i.e., ISI of 0.3125 s between consecutive cues). The frequencies were selected below and above the expected cut‐off frequency (around 2 Hz) for either discrete or continuous movement processing (Stegemöller et al. 2016; Toma et al. 2002). Conditions of 1 and 3.2 Hz will be referred to as ‘1Hz’ and ‘3Hz’, respectively.

The effect of cue modality was evaluated by presenting either auditory or visual cues. The auditory cue consisted of a tone of 0.15 s (pitch A5, i.e., 880 Hz), with fade‐in and fade‐out slopes of 0.05 ms to prevent artefacts like spectral splatter or abrupt changes that might distort the auditory perception. The visual cues consisted of a flashing white circle, presented at the centre of the black background screen for half the ISI duration (i.e., 0.5 and 0.156 s for cues presented at 1 and 3.2 Hz, respectively). Note that cue duration was different between cue modalities. Auditory and visual conditions will be referred to as ‘AUD’ and ‘VIS’, respectively.

The effect of cue rhythmicity was evaluated by presenting either isorhythmic or polyrhythmic cues. Isorhythmic cues consisted of a single, main rhythm, while polyrhythmic cues consisted of two rhythms: the main rhythm and a simultaneously presented faster secondary rhythm in a [2:3] frequency relationship, respectively. Participants were instructed to tap at the main rhythm only. In auditory‐polyrhythmic conditions, the main tone (A5, i.e., 880 Hz) was accompanied by a secondary tone presented at a lower pitch (pitch D3, i.e., 146 Hz). In visual‐isorhythmic conditions, a single white flashing circle was presented in the centre of the screen (visual angle of 5.7°) without a fixation point. In visual‐polyrhythmic conditions, two white, flashing circles were presented left and right of a central, red fixation point (visual angle of 2.8°, distance between two circles 5.7°). Participants were instructed to tap at the rhythm of the left circle (i.e., the main rhythm) while fixating their eyes on the red fixation point in between the two flashing circles. Isorhythmic and polyrhythmic conditions will be referred to as ‘ISO’ and ‘POLY’, respectively.

Due to a minor error in the experimental code, the cue frequency for the condition with fast auditory polyrhythmic cues (i.e., ‘AUD‐POLY‐3Hz’) was set to 3.0 Hz instead of the intended 3.2 Hz for all participants. We do not expect that this influenced the results.

Each cue condition started with a 15‐s resting‐state baseline period, followed by five sequences of finger tapping, of approximately 16 s each, with a short break of 5 s in between sequences. During the baseline period, participants were asked to sit quietly and fixate their eyes on the black screen. Within one sequence of finger tapping, the condition‐specific cue was presented either 16 or 51 times for 1‐ and 3‐Hz conditions, respectively. Participants tapped with their index finger following the rhythm of the main beat. The order of cue conditions was randomized between participants. The MATLAB toolbox, Psychtoolbox‐3 (Brainard 1997), was used for the presentation of cues.

### 2.3 Data Acquisition

Electrical brain activity was measured using an EEG system (waveguard original, ANT Neuro b.v., Hengelo, the Netherlands) with 32 silver/silver chloride (Ag/AgCl) electrodes, positioned according to the 10–10 international system. The ground and reference electrodes were located at the positions AFz and CPz, respectively. By application of conductive gel, the electrode‐skin impedance was aimed to be below 20 kΩ. The finger taps were recorded using a three‐dimensional accelerometer (XS‐354, ANT Neuro b.v., Hengelo, the Netherlands), positioned at the dorsum of the dominant hand, underneath the head of the metacarpal bone of the index finger. Both data from the EEG system and the accelerometer were recorded synchronously at a sampling frequency of 1024 Hz, using the eego amplifier (EE‐225, ANT Neuro b.v., Hengelo, the Netherlands) and the software (eego software v1.9.1., ANT Neuro b.v., Hengelo, the Netherlands).

### 2.4 Data Analysis

Finger taps were recorded using an accelerometer and were used (1) to eliminate sequences that were not performed correctly by comparing the number of finger taps relative to the number of cues (i.e., the intended number of finger taps); (2) to assess the performance of SMS within the different cue conditions; and (3) to segment the EEG data into epochs centred around the occurrences of the taps. The EEG data were used to discern differences in the cortical oscillatory activity of the beta band (12–30 Hz) during finger tapping. The data were analysed with hierarchical Bayesian models to estimate the differences between cue conditions in movement‐related cortical activity and the performance of SMS. Data preprocessing and analysis were executed using MATLAB (version R2023a). The open‐source toolbox EEGLAB was used for the preprocessing and visualization of the data (Delorme and Makeig 2004).

### 2.5 Accelerometer Data

**FIGURE 2:**
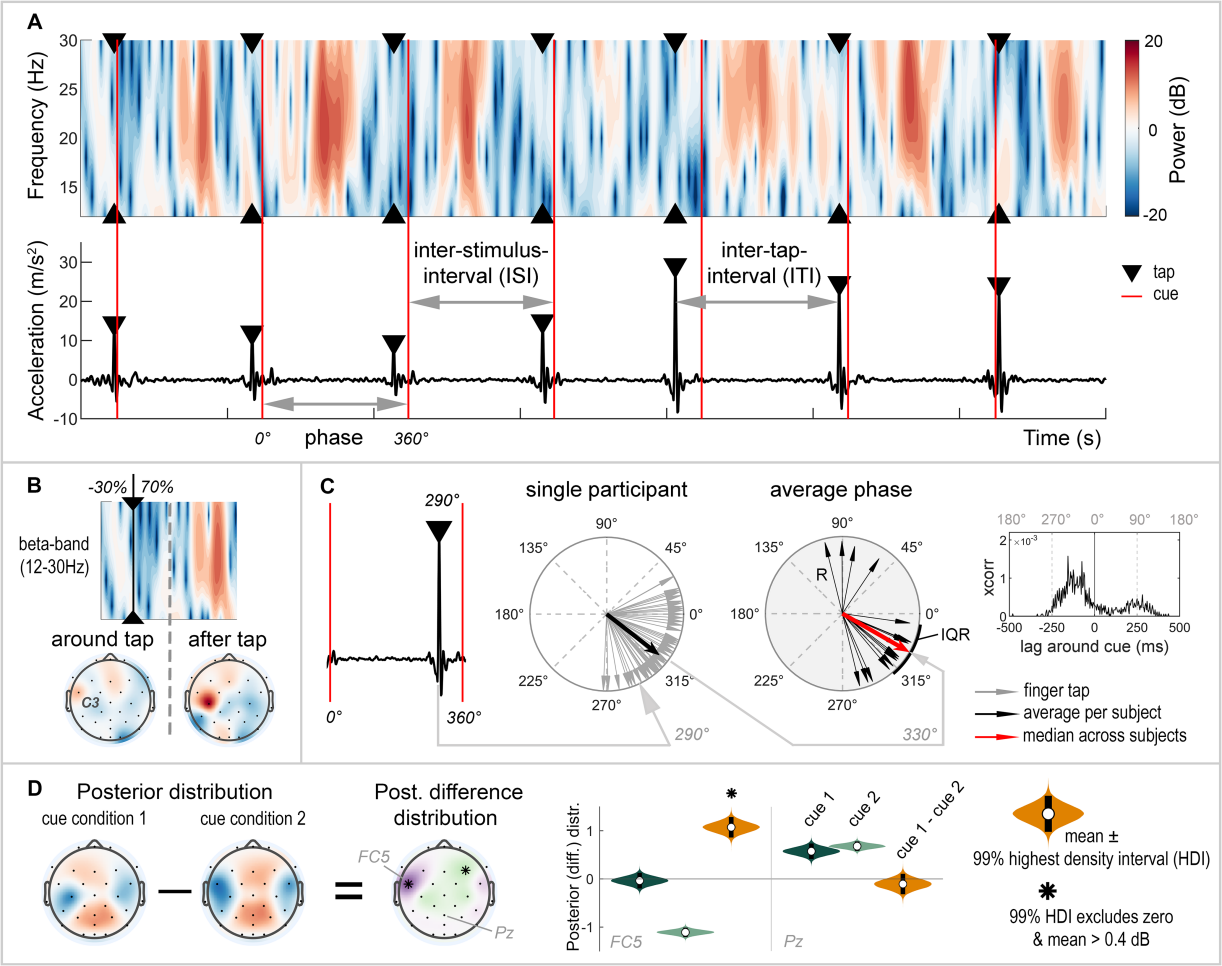
Overview of the data and analyses. Data for a typical participant are shown as an example. (A) Top: Beta band activity (12–30 Hz) recorded from the C3 electrode, showing decreases (blue) and increases (red) in beta power (in decibels) during finger tapping relative to the resting‐state baseline. Bottom: Acceleration data were measured to detect finger taps (black triangles) that were used to divide the EEG data into epochs (see B) and to evaluate the performance of SMS (see A, C). The inter–tap interval (ITI) is the time between two consecutive finger taps. The ISI marks the time between two cues (red lines). (B) Beta activity is shown for a single epoch around a finger tap. Epochs were defined by the ISI, with 30% of the epoch before the tap and 70% after the tap. Epochs were further divided into two time windows of interest: around the tap and after the tap. For each time window, the topographical distribution of the beta power across all electrodes was generated to evaluate the spatial distribution of beta activity across the scalp. (C) The phase (in degrees) represents the timing of the finger tap relative to the preceding and successive cue, expressed on a circular scale from 0° to 360° between two consecutive cues. Polar plots display the phase of individual finger taps (grey arrows) for one participant, the participant's average phase across taps (black arrows, with resultant vector length [R]), the circular median phase across participants (red arrow) and the interquartile range (IQR) across participants (black line surrounding polar plot). The cross‐correlation (xcorr; *right*) presents the lag of the finger tap *relative to* the cue over time (ms). (D) Hierarchical Bayesian models modelled the mean beta power for each electrode and cue condition, providing the posterior distribution. Differences between two cue conditions were assessed by calculating the posterior difference distribution, obtained by subtracting the posterior distributions of the respective cue conditions. The posterior distribution (green violins) and the posterior difference distribution (orange violin) are described by their mean and 99% highest density interval (99%‐HDI). A difference in beta power between cue conditions at a given electrode was considered significant if the 99%‐HDI of the posterior difference distribution excluded zero and the absolute mean exceeded 0.4 dB.

The magnitude of the three‐dimensional acceleration data was calculated by taking the L2 norm. The accelerometer signal was filtered with a zero‐phase, second‐order Butterworth bandpass filter with cut‐off frequencies set at 0.5–70 Hz and down‐sampled to 256 Hz. Finger taps were detected using a sliding window with a size equal to one ISI. Within this window, the highest peak was identified as a finger tap, and the time of the finger tap was extracted (Figure 2A, bottom). The first window was centred around the first cue of the tap sequence (window [cue − 0.5 × ISI : cue + 0.5 × ISI]). If no peak was detected within this window, the subsequent window was centred around the next cue. However, when a finger tap was detected, consecutive windows were positioned relative to the last detected tap, with a shift of half a window length (window [tap + 0.5 × ISI : tap + 1.5 × ISI]). Occasional misidentifications or failures to detect taps, arising from irregular or inaccurate tapping speeds, were corrected through visual inspection. The acceleration data, measured in millivolts, was converted to metres per square second using the calibration points provided by the eego recording software.

#### 2.5.1 SMS Performance

We quantified the accuracy and variability of SMS by determining the discrepancy and coefficient of variation of the inter–tap interval (ITI). The ITI represents the time between two consecutive finger taps (Figure 2A). The discrepancy of ITI (ITI‐D), calculated as the ITI divided by ISI of the cue (Toma et al. 2002), indicates whether the participant tapped at the intended speed as marked by the frequency of the cue (ITI‐D equals one), or tapped too slowly or too fast (ITI‐D above or below one, respectively). Additionally, the coefficient of variation of the ITI (ITI‐CV) indicates whether the participant maintained a constant tapping speed (ITI‐CV equals zero) or tapped at an irregular speed (high ITI‐CV) (Toma et al. 2002). ITI‐CV is calculated as the standard deviation of the ITI divided by its mean, times 100%, to correct for differences in the frequency of the cues.

Whereas ITI‐D and ITI‐CV provide insight into the accuracy and variability of tapping speed, these metrics do not provide information on how participants synchronize with the cues—specifically, whether they tap precisely in time with the cue or tend to tap before or after the cue. The repetitive and periodic nature of the task allowed for the use of a circular scale (0°–360°) to express the occurrence of each finger tap relative to the preceding and successive cue, referred to as the phase (degrees; Figure 2C). The phase is visualized in polar plots, generated using the CircStat (Berens 2009) and CircHist toolbox (Zittrell 2019) in MATLAB, where a finger tap precisely in sync with the cue is represented by an arrow pointing towards 0°. We quantified the participant's average phase across finger taps (black arrows) and the median (red arrow) and interquartile range (IQR) of the phase across participants. The length of the black arrows indicates the resultant vector length (R). This is a measure of the circular spread, demonstrating whether the participant's phases of individual finger taps all point in the same direction (R = 1) or in different directions (R = 0). The use of a circular scale offers some advantages: It is independent of cue frequency, allowing for direct comparisons between 1‐ and 3‐Hz cue conditions. Additionally, it eliminates the need to assume whether participants tap before or after the cue, which is especially challenging when participants tap in antiphase to the cue. Therefore, synchronization to the cue was evaluated by phase differences. For illustrative purposes, we also present the cross‐correlation, showing the lag of the finger tap relative to the cue over time (ms). The cross‐correlation was calculated with a maximum lag equal to half the ISI of the specific cue condition, normalized and corrected for the number of taps.

### 2.6 EEG Data

The EEG data were filtered using a zero‐phase, second‐order Butterworth bandpass filter with cut‐off frequencies set at 1–35 Hz. No artefactual channels, caused by poor electrode‐skin contact, were identified in any of the participants, as the channels' absolute amplitude did not exceed the threshold (> 50 or < 1 μV). Thereafter, independent component analysis was applied to eliminate artefacts attributable to eye blinks or horizontal eye movements (Chaumon et al. 2015). Visual inspection of the independent components was performed to search for the characteristic frontal topographical distribution and the characteristic patterns in the time series, i.e., large deviations in amplitude and step‐like events over time, for eye blinks and horizontal eye movements, respectively. After removal of artefactual independent components, the data were down‐sampled to 256 Hz, and noisy channels were identified through visual inspection of their time series and power spectral density. On average, 1.9 ± 1.0 channels (mean ± SD) out of the 32 per participant were rejected and interpolated using spherical spline interpolation.

Subsequently, the data were re‐referenced using two distinct reference schemes. The average reference (V) was applied for the identification of artefactual epochs. The Laplacian reference (V/m^2^), which acts as a spatial filter and improves the spatial specificity (Kayser and Tenke 2015), was preferred for evaluating changes in power at the electrode level (Pfurtscheller and Lopes da Silva 1999). After the removal of the mastoid electrodes, the data with the average reference and Laplacian reference were stored as two separate datasets.

Time‐frequency analysis was conducted on both datasets using the complex Morlet wavelet convolution, implemented through the spectral multiplication of the EEG data and the complex Morlet wavelets (Cohen 2014). The frequency of the wavelets ranged from 12 to 32 Hz and the number of cycles of the wavelets ranged from 3 to 5 cycles, in 11 logarithmically spaced steps. Power was calculated by taking the square of the absolute wavelet coefficients.

The time series and time‐frequency maps of both datasets were segmented into baseline and condition data. Baseline periods were subdivided into 0.5‐s epochs after excluding the first and last seconds of each 15‐s period. For the cue conditions, epochs with a duration of twice the ISI, centred around the finger tap, were created and grouped according to the specific cue condition.

Artefactual time‐frequency epochs were removed from the baseline and condition data using predefined channel‐specific thresholds. Artefactual epochs were identified based on the dataset with the average reference and later removed from the dataset with the Laplacian reference. An epoch with a potential artefact was detected if (1) the average absolute amplitude (threshold: mean + 2 × SD of the average absolute amplitude), (2) the maximum absolute amplitude (threshold: mean + 10 × SD of the average absolute amplitude), or (3) the maximum power (threshold: mean + 20 × SD of the average power) of one of the channels exceeded the threshold. Next, a visual inspection of the time series was conducted to manually remove any missed artefacts and to check the detection of the potential artefactual epochs.

#### 2.6.1 Beta Oscillations

The movement‐related cortical oscillations of the beta band (12–30 Hz) were calculated using the time‐frequency maps with the Laplacian reference because of its enhanced spatial specificity (Kayser and Tenke 2015). We normalized the beta power, time‐locked to the finger taps, relative to baseline using the decibel transformation (Cohen 2014) to observe increases (red) or decreases (blue) in beta power relative to baseline, referred to as beta enhancement and beta suppression, respectively (Figure 2A). For each cue condition, the common logarithm was taken of the time‐frequency epochs time‐locked to the finger tap, *tap*(*t*, *f*), divided by the frequency‐specific average baseline power across time and epochs, *baseline*(*f*), following Equation (1):

(1) $$\mathrm{POW}\left(t,f\right)=10\;\log_{10}\left(\frac{\mathrm{tap}\left(t,f\right)}{\bar{\text{baseline}\left(f\right)}}\right)$$

with *POW* power (dB), *tap* the time‐frequency amplitude (μV) and *baseline* the average amplitude (μV) across time *t* (s) for each frequency *f* (Hz).

Epochs were shortened to a duration of one ISI, with 30% occurring before the finger tap and 70% after the tap (Figure 2B). This resulted in time windows of −0.3 to 0.7 s and −0.1 to 0.2 s around the finger tap for 1‐ and 3‐Hz conditions, respectively. These epochs were then subdivided into two time windows of interest. Jurkiewicz et al. (2006) reported a beta suppression before the onset and during the execution of a movement, followed by a beta rebound approximately 230 ms after movement completion. Based on this, we divided the epochs into two time windows and calculated the average beta power within these windows of interest (Figure 2B): a window around the tap (−0.3 to 0.2 s for 1‐Hz conditions; relative to 3‐Hz conditions) and a window after the tap (0.2–0.7 s for 1‐Hz conditions; relative to 3‐Hz conditions).

### 2.7 Data Exclusion Criteria

Some participants struggled to adhere to the rhythm of the cues, especially during the first sequences of a cue condition, likely because of the lack of a practice block before the start of the experiment. To address this, we compared the number of finger taps to the number of cues, reflecting the intended number of taps for that sequence. Sequences were excluded from the analysis if the number of finger taps deviated by more than 30% from the number of cues (i.e., the intended number of finger taps; 12–20 taps for the 1‐Hz condition and 36–66 for the 3.2‐Hz condition) as this indicated inattentiveness or non‐adherence to the task. Additionally, if four out of five sequences within a given cue condition were inaccurately performed, the entire cue condition was excluded from subsequent analyses due to the limited amount of available data. Furthermore, the first two taps of each sequence were removed to eliminate potential start‐up effects. Consequently, the percentage of excluded tap sequences across participants was 0% for AUD‐ISO‐1Hz, 4.7% for AUD‐POLY‐1Hz (five sequences from one participant), 0% for AUD‐ISO‐3Hz, 1.2% for AUD‐POLY‐3Hz (one sequence from one participant), 0% for VIS‐ISO‐1Hz, 5.9% for VIS‐POLY‐1Hz (five sequences from one participant), 1.2% for VIS‐ISO‐3Hz and 7.1% for VIS‐POLY‐3Hz (three sequences from two participants). For the remaining sequences, an average of 3.6% ± 1.4% (mean ± SD) artefactual EEG epochs was removed per cue or baseline condition.

### 2.8 Statistical Analysis

#### 2.8.1 Statistical Model

We used a hierarchical Bayesian generalized linear model to evaluate the effect of cue frequency, modality and rhythmicity on the beta oscillatory activity of each electrode and participant (Model 1) and the behaviour of SMS of each participant (Model 2). This approach was used by Kruschke (2015) and extended to include up to five‐way (Model 1) or four‐way interactions (Model 2).

Model 1 modelled the mean power of the beta band, μ, as a linear combination of the effects, β, due to the factors cue frequency (${\vec{x}}_{F}$, two levels: 1‐ and 3‐Hz cues), cue modality (${\vec{x}}_{M}$, two levels: auditory and visual cues), cue rhythmicity (${\vec{x}}_{R}$, two levels: isorhythmic and polyrhythmic cues), EEG electrode (${\vec{x}}_{E}$, 30 electrodes) and participants (${\vec{x}}_{P}$, 17 levels: 17 participants), including all their interactions:

(2) $$\begin{array}{c} \mu_{F,M,R,E,P}=\beta_{0}+\ldots {\vec{\beta }}_{F}{\vec{x}}_{F}+{\vec{\beta }}_{M}{\vec{x}}_{M}+{\vec{\beta }}_{R}{\vec{x}}_{R}+{\vec{\beta }}_{E}{\vec{x}}_{E}\\ \;+{\vec{\beta }}_{P}{\vec{x}}_{P}+\ldots {\vec{\beta }}_{F\times M}{\vec{x}}_{F\times M}+{\vec{\beta }}_{F\times R}{\vec{x}}_{F\times R}\\ \;+{\vec{\beta }}_{F\times R}{\vec{x}}_{F\times R}+{\vec{\beta }}_{F\times E}{\vec{x}}_{F\times E}+{\vec{\beta }}_{F\times P}{\vec{x}}_{F\times P}+\left[\ldots \right]\\ \;+\ldots {\vec{\beta }}_{F\times M\times R}{\vec{x}}_{F\times M\times R}+{\vec{\beta }}_{F\times M\times E}{\vec{x}}_{F\times M\times E}\\ \;+{\vec{\beta }}_{F\times M\times P}{\vec{x}}_{F\times M\times P}+\left[\ldots \right]+\ldots {\vec{\beta }}_{F\times M\times R\times E}{\vec{x}}_{F\times M\times R\times E}\\ \;+{\vec{\beta }}_{F\times M\times R\times P}{\vec{x}}_{F\times M\times R\times P}+\left[\ldots \right]\\ \;+\ldots {\vec{\beta }}_{F\times M\times R\times E\times P}{\vec{x}}_{F\times M\times R\times E\times P} \end{array}$$

with $\beta_{0}$ as the average beta band power across all factors, ${\vec{\beta }}_{a}$ as the main effects due to each factor separately and ${\vec{\beta }}_{a\times b}$, ${\vec{\beta }}_{a\times b\times c}$, ${\vec{\beta }}_{a\times b\times c\times d}$ and ${\vec{\beta }}_{a\times b\times c\times d\times e}$ as all possible interactions between factors. ‘[…]’ indicates the continuation of interaction effects. Note that this model included the effects due to the three experimental manipulations and the effects for each electrode and participant. The statistical model was fitted separately for the beta power around the finger tap and after the finger tap.

Model 2 was the same as Model 1, except it estimated the behavioural outcome measures, ITI‐D and ITI‐CV (fitted separately), and it did not include the factor for EEG electrodes.

#### 2.8.2 Sampling

Posterior distributions of the effects were estimated with Markov Chain Monte Carlo sampling, starting from flat priors. Detailed information on the model specifications, sampling procedure, prior distributions and sampling performance is provided in Data S1.

#### 2.8.3 Estimation

The posterior distribution of each cue condition and electrode was described using the mean and 99% highest density interval (99%‐HDI) to represent central tendency and uncertainty, respectively (Figure 2D). To evaluate differences between two cue conditions, the posterior difference distribution was calculated for each electrode by subtracting the posterior distributions of the respective cue conditions from each other. Here, we report only on differences between cue conditions that were highly likely to be greater or smaller than a null effect. For the beta band power (Model 1), a difference between conditions was considered significant if the 99%‐HDI of the posterior difference distribution excluded 0 dB. Furthermore, to exclude small significant differences, we report only differences where the absolute estimated mean exceeded 0.4 dB. This value was rather arbitrarily chosen but was small considering the largest differences of approximately 1–2 dB. For behavioural measures (Model 2), a significant difference was considered if the 99%‐HDI of the posterior difference distribution excluded 0. Results from edge electrodes on the EEG cap (e.g., Fp1, F7, and Oz) are not considered for significance due to potential artefacts from the Laplacian reference.

We focused on 12 key differences, examining four specific contrasts for each cue characteristic: cue frequency, cue modality and cue rhythmicity. To evaluate the effect of cue frequency, we compared 1‐ and 3‐Hz conditions that had the same cue modality and rhythmicity. These comparisons included AUD‐ISO‐1Hz versus AUD‐ISO‐3Hz, AUD‐POLY‐1Hz versus AUD‐POLY‐3Hz, VIS‐ISO‐1Hz versus VIS‐ISO‐3Hz and VIS‐POLY‐1Hz versus VIS‐POLY‐3Hz. To assess cue modality, we compared auditory and visual conditions while keeping cue frequency and rhythmicity constant. Finally, to evaluate cue rhythmicity, we compared isorhythmic and polyrhythmic conditions that shared the same cue frequency and modality.

#### 2.8.4 Circular Phase

The circular nature of the phase data, and consequently the need for circular priors, makes it challenging to model the phase within a linear Bayesian model. Therefore, a separate statistical analysis was executed on the circular phase data in R (R Team 2020), using the rmTools package (Michel, n.d.). Uniformity of the phase was assessed with the Rayleigh test if the data followed a von Mises distribution (assessed by Watson's test) or Rao's spacing test otherwise. None of the data was found to be uniformly distributed. The nonparametric Moore (1980) test for paired circular data was conducted to evaluate significant differences between cue conditions. The Bonferroni correction was applied to correct for the 12 comparisons (three cue characteristics × four comparisons each), resulting in a significance level of *α* = 0.05/12 = 0.0043.

## 3 Results

Seventeen right‐handed participants were instructed to tap their fingers to the rhythm of various external sensory cues, while the cortical beta band activity was recorded using EEG. First, we quantified how accurately participants synchronized their finger tapping with the various external cues, comparing conditions with slow (1 Hz) and fast (3 Hz) cues, conditions with auditory and visual cues and conditions with simple (isorhythmic) and more complex rhythms (polyrhythmic). Following this, we analysed the corresponding cortical beta band activity related to SMS.

### 3.1 SMS Performance

#### 3.1.1 Tap Accuracy and Consistency

**FIGURE 3:**
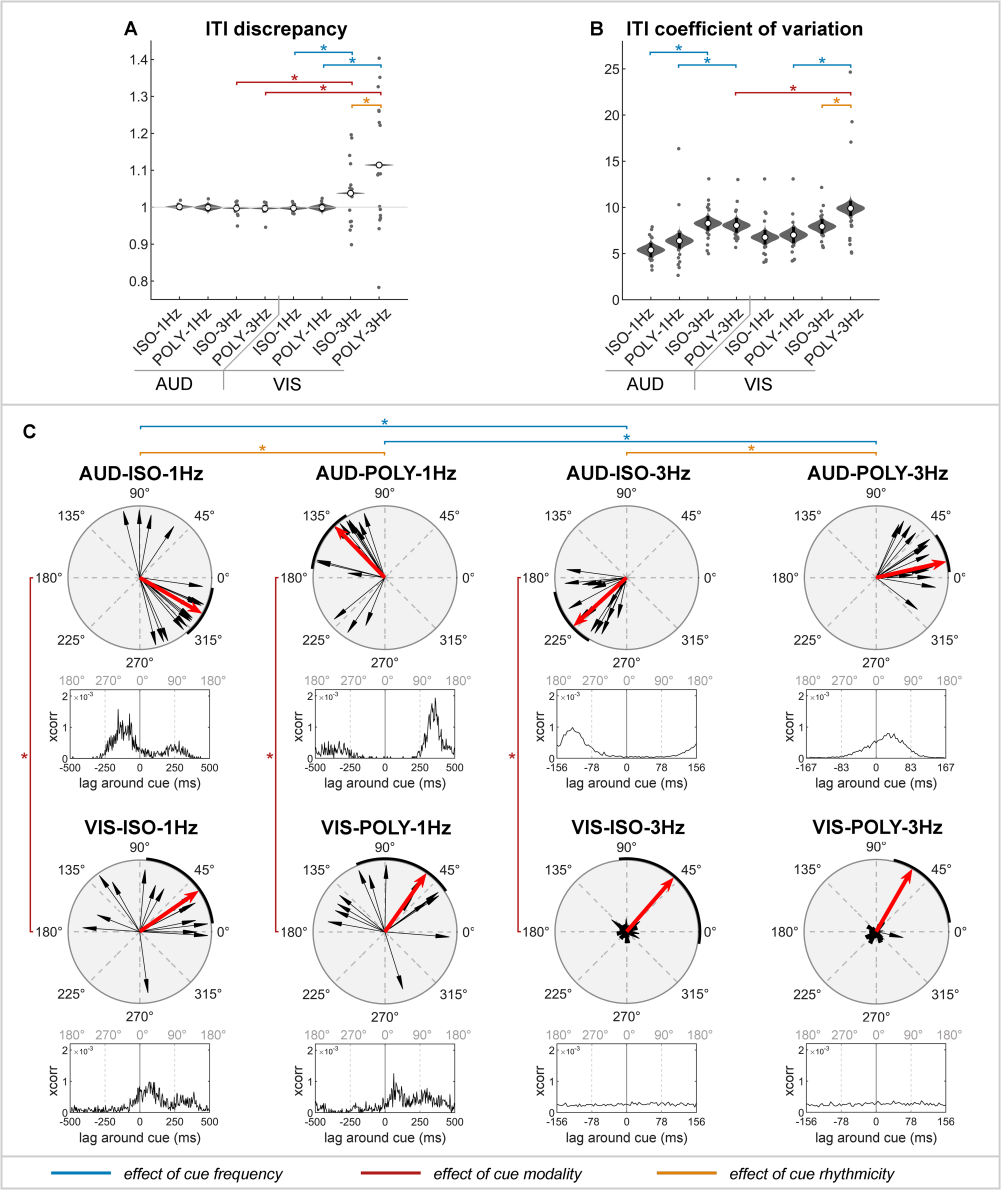
Sensorimotor synchronization behaviour. (A) Discrepancy of the inter–tap interval (ITI‐D) and (B) coefficient of variation of the inter–tap interval (ITI‐CV); violin plots show the posterior distribution and the average measured values for each participant (grey dots). *Comparisons of interests for which 99%‐HDI of the posterior difference distribution excludes zero. (C) Phase (°); arrows represent the average phase per participant (black; resultant vector length R), the median phase across participants (red; unit vector length) and the interquartile range (IQR) across participants (black line surrounding polar plot). *Significant difference in phase after Bonferroni correction (*p* < 0.0043). Cross‐correlation between taps and cues over time.

In auditory conditions, participants accurately tapped at the intended speed (ITI‐D = 1; Figure 3A and Table 1). Neither the frequency nor the rhythmicity affected their ability to maintain the correct speed. However, tapping variability significantly increased with 3‐Hz compared to 1‐Hz auditory cues (ITI‐CV: 1–3 Hz; Figure 3B and Table 2).

**TABLE 1:** Descriptive of the performance measures for each cue condition.

	ITI‐D		ITI‐CV (%)		Phase (°)	
*AUD‐ISO‐1Hz*	1	[0.99 1.01]	5.40%	[4.4 6.4]	330°	(310–352)
*AUD‐POLY‐1Hz*	1	[0.99 1.01]	6.40%	[5.3 7.5]	134°	(122–173)
*AUD‐ISO‐3Hz*	1	[0.99 1.00]	8.30%	[7.3 9.3]	222°	(191–239)
*AUD‐POLY‐3Hz*	1	[0.99 1.00]	8.10%	[7.0 9.1]	13°	(4–36)
*VIS‐ISO‐1Hz*	1	[0.99 1.00]	6.80%	[5.7 7.8]	35°	(7–85)
*VIS‐POLY‐1Hz*	1	[0.98 1.01]	7.00%	[5.9 8.2]	55°	(34–113)
*VIS‐ISO‐3Hz*	1.04	[1.03 1.04]	7.90%	[6.9 9.0]	49°	(96–350)
*VIS‐POLY‐3Hz*	1.11	[1.11 1.12]	9.90%	[8.8 11.0]	60°	(7–76)

*Note:* Values represent the mean (99% highest density interval [99%‐HDI]) of the posterior distribution of the inter–tap interval discrepancy (ITI‐D) and coefficient of variations (ITI‐CV) and the median (interquartile range [IQR]) of the phase (in degrees).

**TABLE 2:** Posterior difference distribution of the performance measures for each comparison of interest.

	ITI‐D		ITI‐CV	
Frequency: 1–3 Hz				
*AUD‐ISO*	0	[0.00 0.01]	−2.90%	[−4.3 −1.4]^a^
*AUD‐POLY*	0	[−0.01 0.02]	−1.70%	[−3.2 −0.1]^a^
*VIS‐ISO*	−0.04	[−0.05 −0.03]^a^	−1.20%	[−2.6 0.3]
*VIS‐POLY*	−0.12	[−0.13 −0.10]^a^	−2.90%	[−4.5 −1.3]^a^
Modality: AUD–VIS				
*ISO‐1Hz*	0	[−0.01 0.01]	−1.40%	[−2.9 0.1]
*POLY‐1Hz*	0	[−0.02 0.02]	−0.60%	[−2.2 1.0]
*ISO‐3Hz*	−0.04	[−0.05 −0.03]^a^	0.30%	[−1.1 1.8]
*POLY‐3Hz*	−0.12	[−0.12 −0.11]^a^	−1.80%	[−3.4 −0.3]^a^
Rhythmicity: ISO–POLY				
*AUD‐1Hz*	0	[−0.01 0.02]	−1.00%	[−2.5 0.5]
*AUD‐3Hz*	0	[−0.01 0.01]	0.20%	[−1.2 1.7]
*VIS‐1Hz*	0	[−0.02 0.02]	−0.20%	[−1.8 1.3]
*VIS‐3Hz*	−0.08	[−0.08 −0.07]^a^	−2.00%	[−3.5 −0.5]^a^

*Note:* Values represent the mean (99% highest density interval [99%‐HDI]) of the posterior difference distribution. Inter‐tap‐interval discrepancy (ITI‐D); Inter‐tap‐interval coefficient of variation (ITI‐CV).

^a^ Significant comparisons of interest for which the 99%‐HDI of the posterior difference distribution excludes zero.

In visual conditions, participants tapped at the intended speed with 1‐Hz cues but tapped too slowly with 3‐Hz cues (ITI‐D: 1–3 Hz; Figure 3A and Table 2). This significant reduction in tap accuracy with 3‐Hz cues was evident for both isorhythmic and polyrhythmic cues but was more pronounced with polyrhythmic cues (ITI‐D: ISO–POLY; Figure 3A and Table 2). Maintaining a consistent tapping speed was particularly challenging with visual, polyrhythmic cues presented at 3 Hz, resulting in a significantly increased tapping variability across modality, rhythmicity and frequency conditions (ITI‐CV: AUD–VIS; Figure 3B and Table 2).

In summary, in auditory conditions, participants accurately synchronized with the speed of both slow and fast cues, although tap variability was greater with 3‐Hz compared to 1‐Hz cues. In visual conditions, participants tapped too slowly with 3‐Hz cues and struggled to maintain a consistent tapping speed with fast, polyrhythmic cues.

#### 3.1.2 Tap Phase

In auditory conditions, a distinct phase difference of approximately half a cycle was observed between isorhythmic and polyrhythmic cues (Figure 3C and Table 1; *p* < 0.001). Specifically, finger taps preceded the occurrence of isorhythmic cues, called negative mean asynchrony (NMA), while taps followed polyrhythmic cues. The NMA was significantly smaller for 1‐Hz isorhythmic cues (median [IQR]: 330° [310°–352°], i.e., −83 ms) than for 3‐Hz isorhythmic cues (222° [191°–239°], i.e., −119 ms; *p* < 0.001). Conversely, delays with polyrhythmic cues were significantly larger for 1‐Hz cues (134° [122°–173°], i.e., 373 ms) than for 3‐Hz cues (13° [4°–36°], i.e., 11 ms; *p* < 0.001).

In visual conditions at 1 Hz, participants tapped after the cue with both isorhythmic and polyrhythmic cues (*p* = 0.104). However, in fast visual conditions, they did not tap at the correct frequency as indicated by the cue (Figure 3A and Table 1), resulting in finger taps that were not phase‐locked to the cue onset (Figure 3C; VIS‐ISO‐3Hz and VIS‐POLY‐3Hz). This led to high within‐participant variability and made the average phase invalid for meaningful comparisons. Consequently, we will not interpret the phase results for comparisons of interest involving fast visual cues. Note that the EEG data analysis remains valid for these conditions, as it is time‐locked to the tap onset rather than cue onset. This approach ensures an accurate interpretation of neural activity related to finger tapping.

In summary, in auditory conditions, a distinct phase difference of half a cycle was observed between isorhythmic and polyrhythmic cues. In fast visual cues, the phase relative to the cue was highly variable within‐ and between‐participants because participants were not able to tap at the intended speed.

### 3.2 Beta Oscillations

**FIGURE 4:**
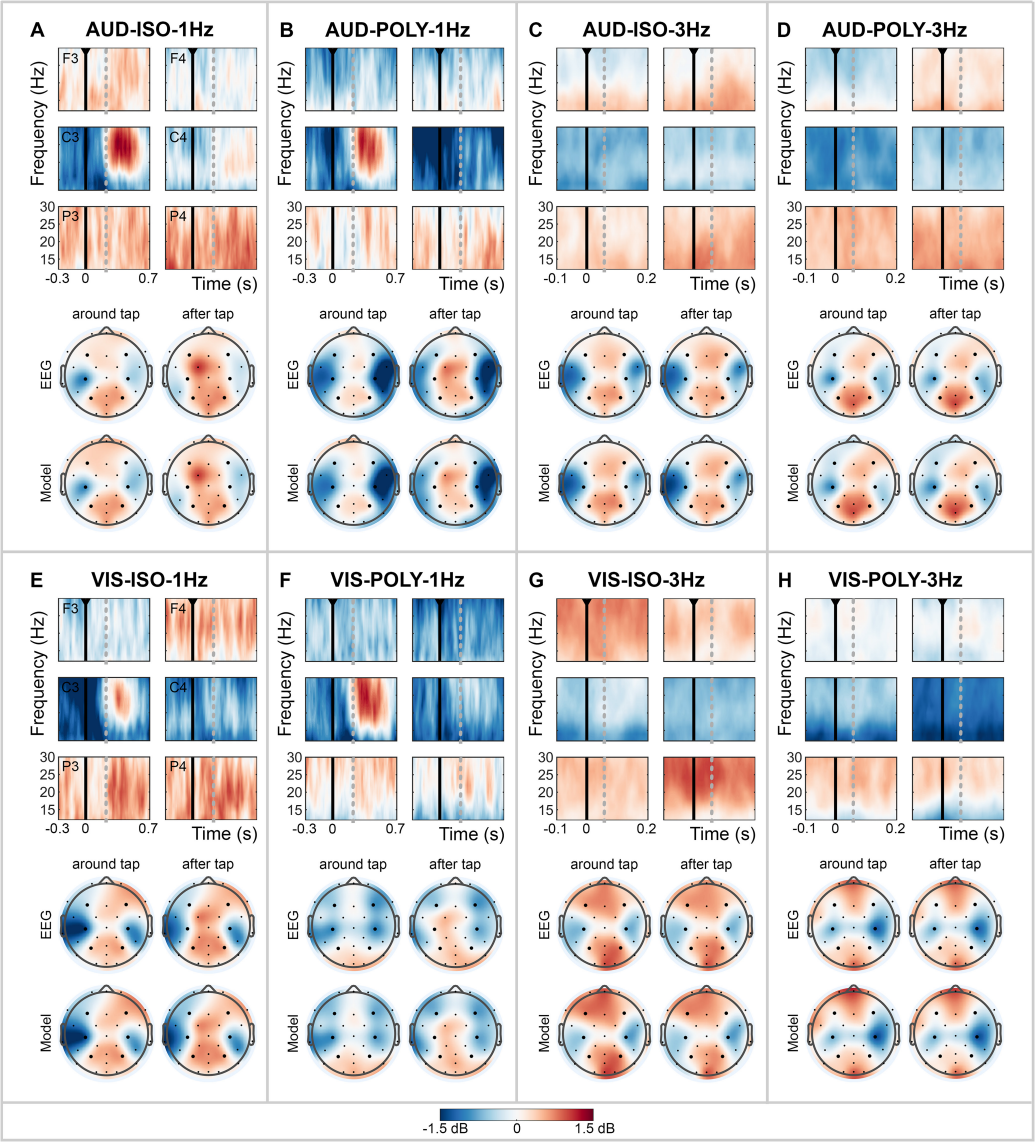
Power of the beta band (dB) during finger tapping guided various cues: (A) AUD‐ISO‐1Hz; (B) AUD‐POLY‐1Hz; (C) AUD‐ISO‐3Hz; (D) AUD‐POLY‐3Hz; (E) VIS‐ISO‐1Hz; (F) VIS‐POLY‐1Hz; (G) VIS‐ISO‐3Hz; (H) VIS‐POLY‐3Hz. (top) The time‐frequency map of the beta band around the finger tap (black lines) is shown for the left and right frontal (F3 and F4), central (C3 and C3) and parietal electrodes (P3 and P4). The map is segmented into two windows: around the tap and after the tap (dotted grey line). (bottom) The topographical distribution of the measured data (EEG) and the posterior distributions (Model; estimated by hierarchical Bayesian model) are shown for both time windows (around tap, after tap).

Time‐frequency maps were calculated for each cue condition to represent changes in the beta power, time‐locked to the finger tap (expressed in decibels; Figure 4), allowing for valid comparisons across conditions regardless of SMS performance. Additionally, scalp distributions of the average beta band are shown for the two time windows: around the finger tap and after the finger tap. The topographical distribution of the data estimated by the hierarchical Bayesian model (Model) closely mirrored the distribution of the measured data (EEG), demonstrating a good model performance (Figure 4; see Data S1 for further details and visualizations on the sampling performance).

Across cue conditions, the beta oscillations, time‐locked to the finger taps, showed a consistent topographical distribution. This distribution was characterized by beta enhancement in midline electrodes over frontal and parietal cortices and beta suppression in lateral central electrodes (C3/C4) extending towards frontocentral and centroparietal electrodes (FC5/FC6, CP5/CP6), covering the sensorimotor, premotor and lateral parietal cortices. We observed differences in the strength and spread of the beta power across the scalp depending on the characteristics of the cue.

**FIGURE 5:**
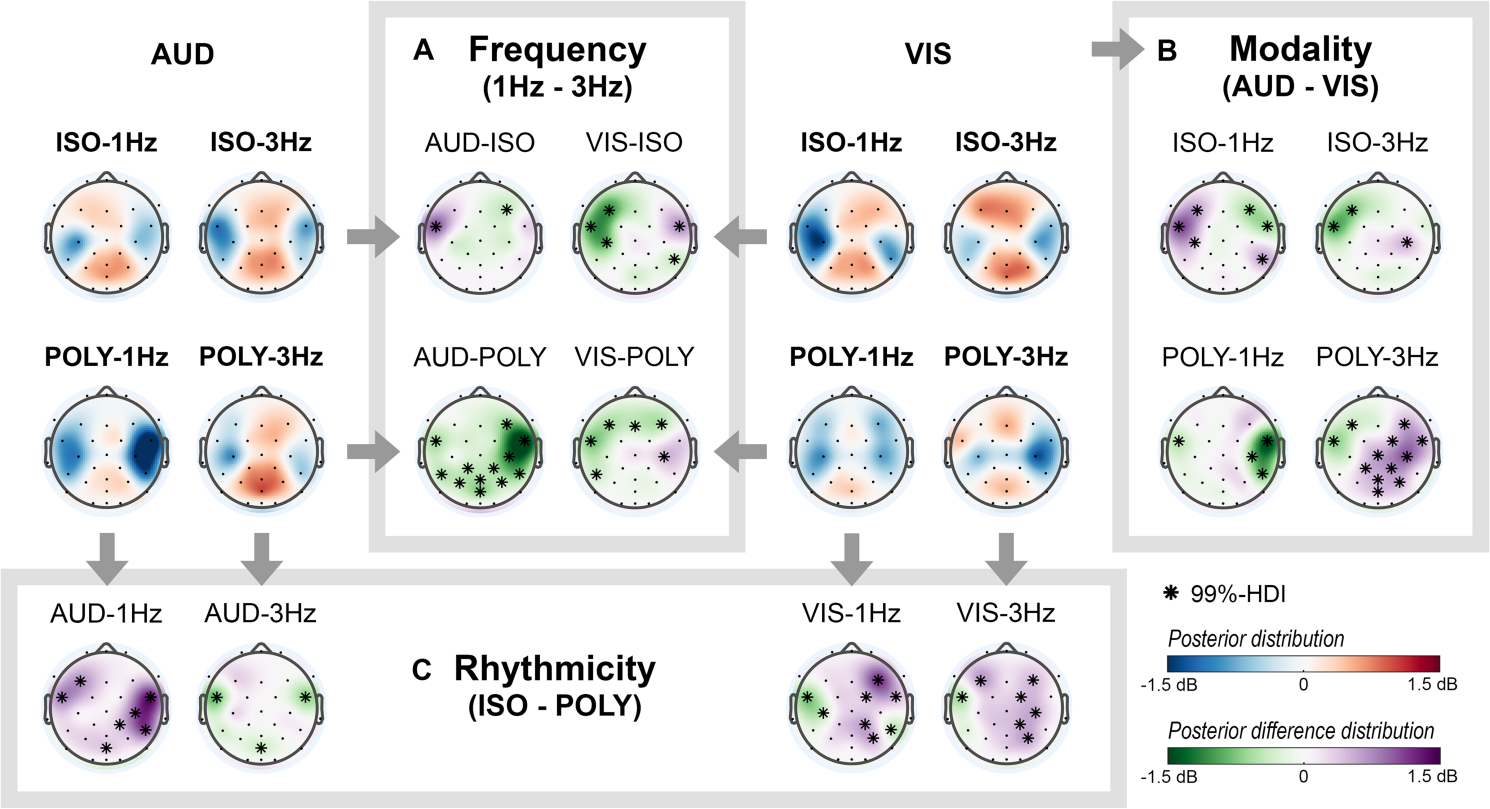
The posterior distribution (blue/red) of the beta band activity around the finger tap for all cue conditions and the posterior difference distributions (green/purple) of the beta band around the finger tap for all comparisons of interest: (A) The effect of cue frequency (1–3‐Hz cue conditions); (B) the effect of cue modality (auditory–visual cue conditions); (C) the effect of cue rhythmicity (isorhythmic–polyrhythmic cue conditions). *Significant electrode positions for which 99%‐HDI of the posterior difference distribution excluded 0 dB, and the absolute mean exceeded 0.4 dB.

Subsequent sections present the scalp topographies of the posterior distributions for each cue condition (red/blue) and the posterior difference distribution (green/purple) of comparisons of interest (Figure 5). Differences in beta power between two cue conditions were significant if the 99%‐HDI of the electrode's posterior difference distribution excluded zero and its absolute mean exceeded 0.4 dB. Significant electrode positions are marked with asterisks in the scalp distributions and are described in the text by their mean and [99%‐HDI]. For regions with multiple significant electrodes, the electrode with the largest effect size is reported in the text.

First, we describe differences in beta band modulations over time in response to cue characteristics. Next, we focus on changes in the scalp distribution of the beta band, in response to cue frequency, cue modality and cue rhythmicity. As statistical results were consistent for the time windows around and after the tap for comparisons regarding cue modality and cue rhythmicity, we report only the results observed around the tap. Complete tables of the results for all electrodes, comparisons and both time windows are provided in Data S2.

#### 3.2.1 Beta Modulation Over Time

The time‐frequency maps reveal changes in beta power time‐locked to the finger tap, particularly at the left central electrode, C3 (Figure 4). With 1‐Hz cues, this electrode showed a distinct beta suppression before and during the execution of the tap, followed by a strong beta enhancement, i.e., beta rebound, after the finger tap (Figure 4A,B,E,F). In contrast, with 3‐Hz cues, a consistent beta suppression over time was observed (Figure 4C,D,G,H).

We did not statistically compare the time windows around and after the tap with each other. However, after the tap, significant differences between 1‐ and 3‐Hz conditions were observed for electrodes covering the left motor cortex. Specifically, 1‐Hz cues elicited beta enhancement, i.e., a beta rebound, after the tap, while 3‐Hz cues induced beta suppression (see Data S2 and Figure S2.1). This difference in response to cue frequency after the tap (1–3 Hz) was evident irrespective of cue modality or rhythmicity: AUD‐ISO (e.g., FC5: 1.14 dB [99%‐HDI: 0.85 1.43]), AUD‐POLY (e.g., C3: 0.94 dB [0.56 1.33]), VIS‐ISO (Cz: 0.53 dB [0.23 0.84]) and VIS‐POLY (e.g., C3: 0.71 dB [0.32 1.10]).

For other electrodes, temporal modulations in beta band activity, time‐locked to the finger tap, were minimal. Moreover, significant differences between cue conditions were consistent between the time windows around and after the tap.

#### 3.2.2 Beta Activity Across the Scalp: 1‐ vs. 3‐Hz Cues

In the left hemisphere, beta suppression around the tap was stronger with 1‐Hz cues compared to 3‐Hz cues in electrodes above the left (pre)motor cortex (FC5, C3, CP5; Figure 5A). This effect was most prominent in the left frontocentral electrode (i.e., FC5) and significant across multiple conditions: AUD‐POLY (−0.63 dB [99%‐HDI: −1.02 to 0.25]), VIS‐ISO (−1.01 dB [−1.29 to 0.72]) and VIS‐POLY (−0.81 dB [−1.20 to 0.42]). However, the opposite effect was observed for AUD‐ISO (FC5: 1.07 dB [0.79 1.36]), showing a stronger beta suppression around the tap for 3‐Hz cues.

In the frontal cortex, beta enhancement around the tap was stronger with 3‐Hz cues compared to 1‐Hz cues (Figure 5A). The stronger beta enhancement in frontal electrodes for 3‐Hz cues was primarily evident in the right hemisphere in auditory conditions (AUD‐ISO, F4: −0.45 dB [99%‐HDI: −0.74 to 0.17]; AUD‐POLY, F4: −0.75 dB [−1.13 to 0.37]) and in the left or both hemispheres for visual conditions (VIS‐ISO, F3: −1.13 dB [−1.41 to 0.84]; VIS‐POLY, e.g., F3: −0.60 dB [−0.99 to 0.21]).

In the right motor cortex and parietal cortex, the effect of cue frequency on beta activity was not consistent across frequency comparisons (Figure 5A). For AUD‐POLY conditions, 1‐Hz cues resulted in a substantially stronger beta suppression in the right motor cortex (e.g., FC6: −1.79 dB [99%‐HDI: −2.17 to 1.41]) and a reduced beta enhancement in the parietal cortex (e.g., POz: −0.75 dB [−1.13 to 0.37]) compared to 3‐Hz cues. However, this effect was likely driven by differences in cue rhythmicity (AUD‐ISO‐1Hz vs. AUD‐POLY‐1Hz), as an increased beta suppression of the right motor cortex and a decreased beta enhancement of the parietal cortex for polyrhythmic cues were consistent across multiple rhythmicity comparisons (Figure 5C, discussed later).

To summarize, tapping with 1‐Hz cues induced a stronger beta suppression around the tap in the left motor cortex, whereas tapping with 3‐Hz cues resulted in a stronger beta enhancement in the frontal cortex. Differences in electrodes above the right motor cortex and parietal cortex were not consistent across cue frequency comparisons.

#### 3.2.3 Beta Activity Across the Scalp: Auditory vs. Visual Cues

The differences between auditory and visual cues were consistent in the time windows around and after the tap (see Data S2 for the results after the tap). Therefore, differences in scalp distribution of the beta band in response to cue modality are reported only for the time window around the tap.

In the left hemisphere, beta suppression was stronger in electrodes above the (pre)motor and frontal cortex (FC5 and F3, respectively) with auditory compared to visual cues (Figure 5B). This effect was most prominent in the left frontocentral electrode (i.e., FC5) and significant across multiple conditions: POLY‐1Hz (−0.51 dB [99%‐HDI: −1.00 to 0.02]), ISO‐3Hz (−0.98 dB [−1.19 to 0.77]) and POLY‐3Hz (−0.68 dB [−0.90 to 0.47]). However, the opposite effect was observed for ISO‐1Hz (e.g., FC5: 1.10 dB [0.76 1.44]), showing a stronger beta suppression with visual cues.

In the right hemisphere, the effect of cue modality was not consistent across conditions (Figure 5B). Specifically, large but opposite differences in beta activity were observed in the right hemisphere for POLY‐1Hz and POLY‐3Hz conditions. In POLY‐1Hz conditions, a stronger beta suppression in electrodes covering the right (pre)motor cortex was observed for auditory compared to visual cues (e.g., FC6: −1.50 dB [99%‐HDI: −2.00 to 1.01]). Conversely, in POLY‐3Hz conditions, beta suppression in the right motor cortex was stronger for visual cues (e.g., C4: 1.02 dB [0.80 1.24]).

In the parietal cortex, differences between auditory and visual cues were observed for POLY‐3Hz conditions only, showing a stronger beta enhancement for auditory compared to visual cues (e.g., P4: 0.53 dB [99%‐HDI: 0.31 0.75]; Figure 5B).

To summarize, tapping guided by auditory cues induced a stronger beta suppression in the left (pre)motor and frontal cortex compared to visual cues, with the exception of ISO‐1Hz conditions. In the right motor cortex, strong but opposite differences between auditory and visual cues were found for POLY‐1Hz and POLY‐3Hz conditions.

#### 3.2.4 Beta Activity Across the Scalp: Isorhythmic vs. Polyrhythmic Cues

The differences between isorhythmic and polyrhythmic cues were consistent in the time windows around and after the tap (see Data S2 for the results after the tap). Therefore, differences in scalp distribution of the beta band in response to cue rhythmicity are reported only for the time window around the tap.

In the left hemisphere, beta suppression was stronger in the (pre)motor cortex, specifically electrode FC5, with isorhythmic compared to polyrhythmic cues (Figure 5C). This effect was significant across multiple conditions: AUD‐3Hz (−0.87 dB [99%‐HDI: −1.01 to 0.73]), VIS‐1Hz (−0.77 dB [−1.11 to 0.43]) and VIS‐3Hz (−0.57 dB [−0.73 to 0.42]). However, the opposite effect was observed for AUD‐1Hz (e.g., FC5: 0.84 dB [0.50 1.18]), showing a stronger beta suppression with polyrhythmic cues.

In the right hemisphere, large and widespread differences in beta activity between isorhythmic and polyrhythmic cues were found, especially for AUD‐1Hz, VIS‐1Hz and VIS‐3Hz conditions (Figure 5C). With polyrhythmic cues, the beta suppression in the right (pre)motor cortex was stronger: AUD‐1Hz (e.g., FC6: 1.36 dB [99%‐HDI: 1.02 1.70]), VIS‐1Hz (FC6: 0.52 dB [0.18 0.87]) and VIS‐3Hz (C4: 0.57 dB [0.41 0.72]). Additionally, in visual conditions, this beta suppression with polyrhythmic cues extended into electrodes covering frontal and parietal areas (frontal, frontocentral, frontoparietal and parietal electrodes), whereas isorhythmic cues demonstrated beta enhancement in these areas: VIS‐1Hz (e.g., F4: 1.22 dB [0.88 1.56]; e.g., P4: 0.57 dB [0.22 0.91]) and VIS‐3Hz (e.g., F4: 0.49 dB [0.34 0.65]; e.g., P4: 0.64 dB [0.48 0.79]). Note that in AUD‐3Hz conditions, this effect was not observed.

To summarize, a consistent effect of cue rhythmicity was observed across comparisons of interest. Isorhythmic cues induced a stronger beta suppression in the electrodes covering the left (pre)motor areas (except for AUD‐1Hz conditions) and showed beta enhancement in the frontal and parietal electrodes. Conversely, polyrhythmic cues induced a stronger beta suppression in the right hemisphere, covering the (pre)motor areas and extending towards the right frontal and parietal electrodes, in visual conditions.

## 4 Discussion

The current EEG study examined the influence of various cue characteristics on finger tapping behaviour and the movement‐related beta band activity associated with SMS, assessing the effect of cue frequency, sensory modality and rhythmicity. The findings showed that participants were able to accurately synchronize with the rhythm of most cues, except for fast visual cues where tapping was performed too slowly. Additionally, tapping variability increased with faster cues, and auditory polyrhythmic cues resulted in a half‐cycle phase delay as compared to auditory isorhythmic cues.

The oscillatory beta band activity, time‐locked to the finger taps, remained relatively constant over time in most brain areas, except for the contralateral sensorimotor cortex, where cue frequency strongly modulated the beta oscillations, consistent with the literature (Stegemöller et al. 2016, 2017; Toma et al. 2002). Across the various cue conditions, the topographical distribution of beta oscillations showed a consistent pattern of beta suppression in the lateral electrodes covering the (pre)motor cortices and beta enhancement in electrodes covering the central frontal and parietal cortices. We observed differences in the strength and spread of the beta power across the scalp depending on the cue characteristics.

We will discuss below how cue characteristics influence tap behaviour and the cortical activity of the beta band. Our findings emphasize the role of cue characteristics in shaping both motor behaviour and the underlying neural processes within and beyond motor areas, suggesting distinct strategies for movement processing and control depending on cue frequency, sensory modality and rhythmicity.

### 4.1 Discrete Movement Processing With Slow Cues Facilitates More Consistent Synchronization Than Continuous Movement Processing With Fast Cues

Participants performed better with 1‐Hz cues compared to 3‐Hz cues, particularly in visual conditions. With auditory cues, they accurately synchronized their tapping speed with both cue frequencies, although tapping variability increased with faster cues. With visual cues, participants accurately synchronized with the frequency of 1‐Hz cues, whereas they tapped too slowly and with greater variability with 3‐Hz cues. Notably, none of the participants synchronized their tapping rhythm with fast visual cues at 3.2 Hz but tapped too slowly at 3.1 Hz (ITI‐D of 1.03) or 2.9 Hz (ITI‐D of 1.11) with visual isorhythmic and polyrhythmic cues, respectively. This finding emphasizes the challenges associated with synchronizing finger tapping to fast visual cues and supports earlier research suggesting a threshold of approximately 2.2 Hz for successful synchronization to visual cues (Hove et al. 2010; Kolers and Brewster 1985; Repp 2003b). The inability to synchronize with fast visual cues implies that participants may have relied on their own internal rhythm rather than adhering to the stimulus‐driven pace.

Consistent with previous literature on slow and fast movements (Boonstra et al. 2006; Seeber et al. 2016; Stegemöller et al. 2016; Toma et al. 2002), we found that the beta oscillations in the contralateral sensorimotor cortex were modulated by cue frequency and, consequently, movement speed. Specifically, with 1‐Hz cues, beta suppression before and during the execution of a finger tap was followed by a strong beta rebound approximately 200 ms after the tap, indicating the preparation and execution of discrete, individually timed movements (Toma et al. 2002). In contrast, with 3‐Hz cues, a sustained beta suppression was observed, which implies continuous rather than discrete movement processing (Toma et al. 2002). Previous EEG studies have identified a cut‐off point between discrete and continuous movement processing at around 2 Hz (Stegemöller et al. 2016; Toma et al. 2002). This threshold corresponds to the behavioural study of Huys et al. (2008) showing that repetitive, discrete movements are typically feasible up to velocities of 2 Hz, whereas higher speeds result in the execution of continuous movements (Huys et al. 2008). While finger taps were consistently executed as discrete movements across cue conditions, including a stationary phase at the table surface, we propose that at higher cue frequencies, the timing of individual movements was no longer processed distinctly. Instead, the general rhythm of tapping was prioritized. This continuous movement processing may explain the increased tap variability with 3‐Hz cues, as taps are no longer aligned individually with the timing of cues. These differences in beta modulation over time may directly drive the observed variations in tap velocity and performance between 1‐ and 3‐Hz cues, or alternatively, the neural activity could be shaped by the variations in tapping behaviour.

Furthermore, our results revealed that the distribution of beta oscillations varied across the scalp depending on cue frequency. With 1‐Hz cues, we observed stronger beta suppression above the left sensorimotor cortex around the tap that extended further into the premotor and parietal cortices, compared to more localized activity with 3‐Hz cues. The increased activation of the premotor cortex, related to sensory guidance and timing of movements (Chen et al. 2008, 2009; Pollok et al. 2009; Pollok, Rothkegel, et al. 2008), likely facilitated accurate synchronization to slow cues, as positive correlations between activity in the dorsal premotor cortex and tapping accuracy have been reported (Miyata et al. 2022). The increased activation of the parietal cortex likely aided in aligning individual taps with the timing of cues, considering its role in sensorimotor integration and anticipatory motor control by comparing actual movements with the intended motor plan (Krause et al. 2014; Pollok, Gross, et al. 2008; Pollok et al. 2005). For example, beta suppression of the parietal cortex has been observed during gait adaptations in response to cue‐induced changes in step tempo (Wagner et al. 2016). Thus, the allocation of sensorimotor, premotor and parietal areas around the tap, along with more accurate tapping performance with 1‐Hz cues, suggests that temporal information was more easily extracted from slow cues, facilitating more effective sensorimotor integration and error correction, resulting in more accurate alignment of each finger tap to the timing of the cue.

In contrast, with 3‐Hz cues, we observed a stronger beta enhancement around the tap in the frontal cortex. This suggests greater engagement of the frontoparietal network, which is involved in endogenous, top‐down attention and control of behaviour (Buschman and Miller 2007, 2014; Engel and Fries 2010). The frontal cortex plays a critical role in integrating sensory information into internal models via its connections with secondary motor areas, particularly during more complex tasks (Miller and Cohen 2001). For example, enhanced frontal beta power has been observed during fast compared to slow movements (Miller and Cohen 2001; Seeber et al. 2016) and during gait adaptations following cue‐induced changes in step tempo (Wagner et al. 2016). Therefore, the stronger beta enhancement around the tap in the frontal cortex suggests increased cognitive demands to synchronize with fast cues.

In summary, our findings suggest distinct neural mechanisms underlying SMS with slow versus fast cues. The differences in the beta activity in the contralateral sensorimotor cortex over time may reflect discrete and continuous processing of movement timing with 1‐ and 3‐Hz cues, respectively. However, it remains to be determined whether these differences in neural dynamics directly drive differences in motor behaviour, i.e., tapping speed, or are the consequence of it. Additionally, we expect that the allocation of the left motor, premotor and parietal areas with 1‐Hz cues around the execution of the finger tap, facilitated sensorimotor integration and error correction, aligning individual finger taps to the timing of cues. This discrete processing of the timing of finger taps resulted in a consistent and stable tap rhythm when tapping to 1‐Hz cues. With 3‐Hz cues, stronger beta enhancement in frontal areas and increased tap variability may reflect greater endogenous, top‐down control of movement, in which the rhythm instead of the specific timing of the cues is more important. The timing of movements may be processed continuously, resulting in greater tap variability when tapping to 3‐Hz cues.

### 4.2 Enhanced Sensory Guidance With Auditory Cues Leads to More Accurate Synchronization Compared to Visual Cues

Our results showed that finger tapping with auditory cues allows for higher cue frequencies as compared to visual cues, in line with the literature (Hove et al. 2010; Repp 2003b). Participants were unable to synchronize their tapping speed to the rhythm of fast visual cues, whereas tapping to auditory cues was performed accurately for both cue frequencies. Tapping too slowly or too fast naturally resulted in a highly variable phase for the fast visual cues. Consistent with our results, Repp (2003b) reported that differences between auditory and visual cues were not observed in terms of the variability of ITI, but rather in the variability of the phase relative to the cue, and noticed during the experiments that ‘participants rarely attempted to correct their tapping but simply continued to tap “blindly” at a steady state, hoping that the taps would coincide with the sequence events’ (Repp 2003b). Therefore, we expect that participants aligned their finger taps to an internally generated rhythm, rather than the occurrences of the fast visual cues. (Repp 2003b).

Beta band oscillations did not show time‐dependent modulation based on cue modality; similar significant results were found around and after the tap. Therefore, we focus our discussion on differences in scalp distribution of beta activity between auditory and visual cues around the tap. Above the left premotor cortex (electrode FC5), beta suppression was stronger for auditory cues, especially in 3‐Hz conditions. Given the superior tapping performance with fast auditory cues, this suggests that temporal auditory information was more easily integrated into the motor plan and that auditory cues provided better sensory guidance during finger tapping.

In other brain areas, we did not observe a distinct and consistent effect of cue modality across multiple comparisons of interest. We propose that observed differences may be introduced by other cue characteristics, potentially overshadowing any modality‐dependent beta oscillatory networks for SMS, as reported in the literature. A previous EEG study examined predictive beta activity using an omission paradigm with auditory and visual rhythms (Comstock et al. 2021). They found common activations in parietal and frontal cortices but modality‐specific activations in temporoparietal, midline and occipital areas for visual cues and sensorimotor areas for auditory cues. Additionally, MEG and fMRI studies on finger tapping reported conflicting results regarding the role of the dorsal and ventral premotor cortices with auditory and visual cues (Jäncke et al. 2000; Jantzen et al. 2005; Pollok et al. 2009). The present study cannot further elucidate contradicting findings in the literature. The low spatial resolution of EEG and limited electrode coverage on the primary auditory and visual cortices may have contributed to the lack of clear modality‐specific differences in beta band activity in this study.

In summary, our results showed that participants were unable to synchronize with fast visual cues, whereas tapping to auditory cues was performed accurately. The enhanced beta suppression around the left premotor area observed for auditory cues suggests more sensory guidance of movements with auditory compared to visual cues.

### 4.3 Bottom–Up Processing and Subdivision Benefit of Polyrhythms Results in Similar Tap Accuracy Compared to Top‐Down Processing of Isorhythmic Cues

In auditory conditions, cue rhythmicity influenced the phase of tapping relative to the cues, with approximately half a cycle. Our results align with previous studies, showing that distractor tones can strongly affect the timing of tapping relative to the target stimuli (Repp 2003a, 2004). In polyrhythmic tapping tasks without specific instructions, individuals tend to synchronize with the fastest or the lowest pitched rhythm (Handel and Oshinsky 1981), as synchronization to lower pitched tones is more accurate and requires less listening effort (Shivhare and Sanjram 2021; Varlet et al. 2020). Despite instructions in the present study to tap into the slowest, high‐pitched rhythm, the natural tendency to synchronize with the fastest or lowest pitched rhythm (Handel and Oshinsky 1981; Repp 2003a) likely contributed to the phase shift towards the secondary beat with auditory polyrhythmic cues.

Although the secondary rhythm caused a phase delay between isorhythmic and polyrhythmic cues, tapping speed accuracy and variability with auditory polyrhythmic cues remained unaffected by cue rhythmicity. This may be explained by the so‐called subdivision benefit, introduced by the secondary beat (Repp 2003b). Tapping every second event of a faster sequence (1:2 tapping) reduces phase variability compared to tapping every event (1:1 tapping) of a sequence. This benefit has been observed for auditory and visual sequences with inter–beat intervals above 250–200 and 470 ms, respectively (Repp 2003b). We propose that auditory polyrhythms shifted the tapping phase towards the secondary beat due to its rhythm and pitch, while the subdivision benefit of polyrhythms simultaneously facilitated synchronization, resulting in an accurate and consistent tapping rhythm.

With visual cues, the subdivision benefit likely facilitated synchronization to slow polyrhythmic cues, but not too fast visual polyrhythmic cues. The short inter–beat interval for fast polyrhythms may have limited this benefit, reducing tapping accuracy and consistency. Alternatively, the increased tap variability may be due to differences in stimuli presentation, with foveal presentation of isorhythmic cues and peripheral presentation of polyrhythmic cues, potentially causing differences in visual processing time (Staugaard et al. 2016). Therefore, the effect of rhythmicity with visual cues needs further investigation by using either foveal or peripheral cue presentation.

Beta band oscillations did not show time‐dependent modulation based on cue rhythmicity; similar significant results were found around and after the tap. Therefore, we focus our discussion on differences in scalp distribution of beta activity between isorhythmic and polyrhythmic cues around the tap. Our results revealed distinct differences in the distribution of beta oscillations between isorhythmic and polyrhythmic cues. Given that tapping accuracy and variability were comparable for isorhythmic and polyrhythmic cues, we suggest that these differences in beta band activity reflect distinct control strategies or compensatory processes to maintain consistent tap performance.

In general, isorhythmic cues demonstrated a stronger beta enhancement in the frontal and parietal regions, indicating more endogenous, top‐down control of movement. In contrast, polyrhythmic cues, especially at 1 Hz, were characterized by reduced beta enhancement or even beta suppression in these areas, accompanied by strong beta suppression in the right hemisphere extending into the (pre)motor, lateral frontal and parietal regions.

The pronounced beta suppression in the right hemisphere with slow polyrhythms aligns with previous fMRI studies, showing increased activations in the right premotor, prefrontal and inferior parietal cortices, as well as bilateral supplementary motor areas and cerebellum, in response to greater rhythmic complexity (Chen et al. 2008; Thaut et al. 2008). Since motor performance remained unaffected by rhythmic complexity, it has been suggested that the observed differences reflect compensatory processes that adapt to variations in internal processing demands for sensorimotor integration, ensuring a consistent motor output (Thaut et al. 2008).

The right hemisphere plays a crucial role in both timing and visuomotor information processing. Studies on timing processing highlighted the crucial role of the right hemisphere, especially the right dorsolateral prefrontal cortex and the right inferior parietal cortex (Koch et al. 2009; Macar et al. 2002). Similarly, the right hemisphere is more important in visuomotor information processing than the left hemisphere and is considered more ‘visually intelligent’ in a spatial search task (Corballis 2003; Farnè et al. 2003). Therefore, we propose that the enhanced beta suppression in the right hemisphere reflects increased demands on temporal processing and sensorimotor integration posed by the slow polyrhythmic cues to achieve similar tap accuracy as with slow isorhythmic cues.

We further propose that polyrhythmic cues elicit more exogenous, bottom‐up attention and control of movements as suggested by several observations: (1) The reduced or absent beta enhancement in the frontal cortex suggests limited top‐down control of movement. (2) The strong involvement of the right hemisphere may point towards engagement of the ventral attention network, which is associated with exogenous, stimulus‐driven attention (i.e., bottom‐up) in visual tasks, as opposed to the dorsal attention network, which is related to endogenous, goal‐directed attention (i.e., top‐down) (Tang et al. 2016). Although the spatial resolution of the EEG data is insufficient to conclusively differentiate between the dorsal (frontal eye fields, inferior and superior parietal cortices) and ventral areas (temporal–parietal junction, ventral frontal cortex), the scalp distribution of the beta oscillations points more towards the role of the ventral than the dorsal attention network. (3) The half‐cycle phase shift observed with auditory polyrhythmic cues further supports stimulus‐driven, bottom‐up attention and control of movement, as the natural tendency to align taps with the faster, low‐pitched secondary beat (i.e., bottom‐up) prevailed over the internal goal to tap to the primary beat (i.e., top‐down).

In summary, tap accuracy and consistency were comparable between isorhythmic and polyrhythmic cues, likely as a result of the subdivision benefit introduced by the secondary beat of the polyrhythmic cues. Beta enhancement in frontal and parietal areas suggests greater endogenous, top‐down control of movement with isorhythmic cues. In contrast, the widespread beta suppression with polyrhythmic cues (especially 1 Hz) and the phase delay with auditory polyrhythmic cues suggest more stimulus‐driven, bottom‐up control of movement with polyrhythmic cues.

### 4.4 Limitations and Recommendations

Future research should explore the role and direction of beta band modulation in relation to SMS, particularly in regions beyond the commonly studied motor areas. Additionally, methodological limitations in the experimental design should be considered in future studies. First, to obtain a better understanding of the variability between participants, we propose the inclusion of questionnaires that assess participants' musicality (Chen et al. 2009; Repp 2003b; Whitton and Jiang 2023) and their preferences and experiences regarding the various cue conditions. These questionnaires may provide valuable insights into the variability between participants observed in performance measures or cortical activity in certain cue conditions.

Second, additional experimental conditions should be considered to gain insights into the distinct cortical dynamics induced solely by the cues or the motor task. The beta oscillations demonstrated in our results represent a summation, or possibly interaction, of the cortical activity related to both motor processing and sensory processing of the cues. It remains unclear which electrophysiological changes can be attributed to cue‐related motor control. To disentangle these effects, future studies may measure additional conditions with cue presentation during rest and continuation of tapping after the elimination of the sensory cue, as previously done (Boonstra et al. 2006; Jäncke et al. 2000; Jantzen et al. 2005; Konoike et al. 2012; Lewis et al. 2004; Meijer et al. 2016; Pouthas et al. 2005; Rao et al. 1997; Seeber et al. 2016). Furthermore, the repetitive and cyclic nature of the tapping tasks raises the question of whether beta modulation drives or results from the actual tapping behaviour. Neuromodulation techniques, such as transcranial magnetic stimulation, could help clarify the causal relationship between beta band activity and motor output (Pollok, Rothkegel, et al. 2008). However, considering the already substantial number of conditions and subsequently a large number of statistical comparisons, we made the decision not to include these additional conditions in the present study.

Third, the study did not include a formal practice block at the start of the experiment, but only a brief instructional video to introduce the cue conditions. Consequently, some participants found it challenging to synchronize with the rhythm of the cues, particularly during the first sequences of a cue condition. These sequences effectively served as an unintended ‘practice period’ and were therefore excluded from further analyses if the number of taps deviated by more than 30% from the number of cues. This criterion was subjectively chosen as a practical compromise to balance retaining correct sequences and excluding incorrect, practice‐like sequences.

Fourth, differences in the cue presentation must be considered when comparing cue conditions: (1) Auditory (0.15 s) and visual cues (half the ISI) differ in presentation time, with visual cues potentially introducing a binary meter due to their on/off pattern. It is unclear if this introduced a subdivision benefit as discussed above for polyrhythmic cues or how it affects time perception or tapping performance. (2) The foveal presentation of visual isorhythmic cues may differ in visual processing from the peripheral presentation of visual polyrhythmic cues (Staugaard et al. 2016). These differences in stimulus presentation require further investigation. (3) The experimental setup introduced a slight jitter in stimulus presentation, which could be improved in future studies by using specialized screens with higher refresh rates and dedicated audio cards. We do not expect that this minor jitter affected the participant's ability to perceive the rhythms, as none of them reported noticing any irregularities in the stimulus presentation.

Last, the hierarchical Bayesian models used in this study were beneficial because they enabled the implementation of our complex 2 × 2 × 2 study designs and avoided the need for multiple comparisons. The hierarchical Bayesian model could potentially be improved by incorporating prior knowledge of specific locations of the electrodes on the scalp, as suggested by Dimmock et al. (2023). However, introducing another parameter to the already extensive model used in the current study might have made the model too complex and ineffective to estimate the data.

## 5 Conclusion

This exploratory EEG study highlights the importance of cue characteristics in shaping motor behaviour and underlying neural processes within and beyond the motor cortices, suggesting distinct strategies for movement processing and control depending on cue frequency, sensory modality and rhythmicity. Specifically, our findings support previous hypotheses on the existence of separate frequency‐dependent control mechanisms for discrete and continuous movement processing with slow and fast cues, respectively. Auditory cues enable more accurate synchronization compared to visual cues, particularly at higher frequencies, likely due to their stronger sensory guidance. The scalp distribution of beta oscillations suggests a shift from endogenous, top‐down control with isorhythmic cues to more exogenous, bottom‐up control with polyrhythmic cues, especially at slow frequencies. Future research may further elucidate the electrophysiological changes associated with both cue processing and motor control, offering deeper insights into their distinct contributions to SMS in response to various cue characteristics.

## Author Contributions

**Janne J. A. Heijs:** conceptualization, data curation, formal analysis, investigation, methodology, software, supervision, validation, visualization, writing – original draft, writing – review and editing. **Silvana Huertas‐Penen:** conceptualization, data curation, formal analysis, investigation, methodology, software, validation, writing – original draft, writing – review and editing. **Marc M. van Wanrooij:** formal analysis, methodology, software, validation, writing – review and editing. **Bettina C. Schwab:** methodology, writing – review and editing. **Richard J. A. van Wezel:** conceptualization, funding acquisition, methodology, project administration, resources, supervision, writing – review and editing. **Tjitske Heida:** conceptualization, formal analysis, funding acquisition, methodology, project administration, resources, supervision, writing – review and editing.

## Conflicts of Interest

The authors declare no conflicts of interest.

### Peer Review

The peer review history for this article is available at https://www.webofscience.com/api/gateway/wos/peer‐review/10.1111/ejn.70112.

## Supporting information


**Data S1** Supporting information.

## Data Availability

The data that support the findings of this study are openly available in the Data Sharing Collection of the Donders Repository at https://doi.org/10.34973/68nj‐pt92.
