# Supplementary material for: The Effect of Cue Frequency, Modality and Rhythmicity on Finger Tapping Behaviour and Movement‐Related Cortical Activity
Source: Eur J Neurosci. 2025 Apr 19;61(8):e70112. doi: 10.1111/ejn.70112 (PMC12008777; doi:10.1111/ejn.70112)
Supplement: Supplementary file 1 — Data S1 Supporting information. [file EJN-61-0-s001.pdf]

Supplementary materials

Table of Contents

S1. Supplementary methods 1
Hierarchical Bayesian model 1
Markov Chain Monte Carlo (MCMC) sampling 3
Posterior Predictive Check 4
Considerations calculation power 4
References 5
S2. Supplementary results 6

S1. Supplementary methods

Hierarchical Bayesian model

Bayesian models estimate the relative probability of a parameter (i.e., outcome measure of interest) taking on a specific value, considering both measured data and prior expectations (1, 2).

The use of hierarchical Bayesian models in the present study provides several advantages (1, 2). First, they enable the implementation of complex study designs, allowing simultaneous estimation of effects for various cue characteristics, electrodes, and participants. This improves statistical sensitivity and avoids the need for multiple comparison corrections. Second, the hierarchical structure of the model considers that parameters, such as those from individual participants or electrodes, are not entirely independent. Third, these models effectively handle imbalanced datasets, which prevents the exclusion of participants due to missing data from a single cue condition. Fourth, a process called 'shrinkage' reduces the impact of outliers, leading to more accurate estimations of the effects of interest.

We fitted two different Bayesian models to derive the posterior distributions of the outcome variables. Model 1 estimated beta power around the finger tap and beta power after the finger tap. Model 2 estimated the performance measures ITI-D and ITI-CV. Although both models share similarities, Model 2 does not include the factor for EEG electrodes, which was incorporated in Model 1. Below, we provide a detailed explanation of Model 1.

The model

The Bayesian model (Figure S1.1) assumed that the beta power from each epoch (y_i, representing the finger taps) follows a normal distribution with a mean, μ_i, and standard deviation, σ.

$$y_i \sim \mathrm{Normal}(\mu_i, \sigma)$$

The mean beta power, μ_i, was modelled as a linear combination of the predictor factors: cue frequency ($\vec{x}_F$, with 2 levels (f): 1 Hz, 3 Hz cues), cue modality ($\vec{x}_M$, with 2 levels (m): auditory, visual cues), cue rhythmicity ($\vec{x}_R$, with 2 levels (r): isorhythmic, polyrhythmic cues), EEG electrode ($\vec{x}_E$, with 30 levels (e): 30 electrodes), and participants ($\vec{x}_P$, with 17 levels (p): 17 participants).

$$\begin{gathered}
\mu_i = \beta_0 + \cdots \\
\vec{\beta}_F \vec{x}_F + \vec{\beta}_M \vec{x}_M + \vec{\beta}_R \vec{x}_R + \vec{\beta}_E \vec{x}_E + \vec{\beta}_P \vec{x}_P + \cdots \\
\vec{\beta}_{F\times M} \vec{x}_{F\times M} + \vec{\beta}_{F\times R} \vec{x}_{F\times R} + \vec{\beta}_{F\times R} \vec{x}_{F\times R} + \vec{\beta}_{F\times E} \vec{x}_{F\times E} + \vec{\beta}_{F\times P} \vec{x}_{F\times P} + \cdots \\
\vec{\beta}_{M\times R} \vec{x}_{M\times R} + \vec{\beta}_{M\times E} \vec{x}_{M\times E} + \vec{\beta}_{M\times P} \vec{x}_{M\times P} + \cdots \\
\vec{\beta}_{R\times E} \vec{x}_{R\times E} + \vec{\beta}_{R\times P} \vec{x}_{R\times P} + \cdots \\
\vec{\beta}_{E\times P} \vec{x}_{E\times P} + \cdots \\
\vec{\beta}_{F\times M\times R} \vec{x}_{F\times M\times R} + \vec{\beta}_{F\times M\times E} \vec{x}_{F\times M\times E} + \vec{\beta}_{F\times M\times P} \vec{x}_{F\times M\times P} + \cdots \\
\vec{\beta}_{F\times R\times E} \vec{x}_{F\times R\times E} + \vec{\beta}_{F\times R\times P} \vec{x}_{F\times R\times P} + \vec{\beta}_{F\times E\times P} \vec{x}_{F\times E\times P} + \cdots \\
\vec{\beta}_{M\times R\times E} \vec{x}_{M\times R\times E} + \vec{\beta}_{M\times R\times P} \vec{x}_{M\times R\times P} + \vec{\beta}_{M\times E\times P} \vec{x}_{M\times E\times P} + \cdots \\
\vec{\beta}_{R\times E\times P} \vec{x}_{R\times E\times P} + \cdots \\
\vec{\beta}_{F\times M\times R\times E} \vec{x}_{F\times M\times R\times E} + \vec{\beta}_{F\times M\times R\times P} \vec{x}_{F\times M\times R\times P} + \cdots \\
\vec{\beta}_{F\times M\times E\times P} \vec{x}_{F\times M\times E\times P} + \vec{\beta}_{F\times R\times E\times P} \vec{x}_{F\times R\times E\times P} + \cdots \\
\vec{\beta}_{M\times R\times E\times P} \vec{x}_{M\times R\times E\times P} + \cdots \\
\vec{\beta}_{F\times M\times R\times E\times P} \vec{x}_{F\times M\times R\times E\times P}
\end{gathered}$$

The standard deviation, σ, was assigned a gamma prior, with shape (S) and rate (R) parameters defined as half and twice the standard deviation, respectively, across all measured data (i.e., all epochs of all conditions, electrodes, and participants).

$$\sigma \sim \mathrm{Gamma}(S, R)$$

Note that the model standard deviation, σ, is a single posterior distribution which is the same for all factors, while the model mean, μ, differs depending on the factors. The decision to use a fixed standard deviation across all factors was supported by the approximately equal variance of the outcome measures per factor in the measured data.

The baseline, β_0, was assigned a flat prior, namely a normal distribution with the average power, μ_{POW}, as mean, and five times the standard deviation of the power, σ_{POW}, across all measured data.

$$\beta_0 \sim Normal(\mu_{\mathrm{POW}}, 5\sigma_{\mathrm{POW}})$$

The priors for each level of each factor, β_{factor}, were modelled using normal distributions, with a mean of zero and a standard deviation, σ_{factor}, which was assigned a gamma distribution with shape (S) and rate (R) defined as half and twice the standard deviation, σ_{POW}, respectively, across all the measured data.

$$\beta_{factor} \sim Normal(0, \sigma_{\mathrm{factor}})$$

$$\sigma_{\mathrm{factor}} \sim Gamma(S, R)$$

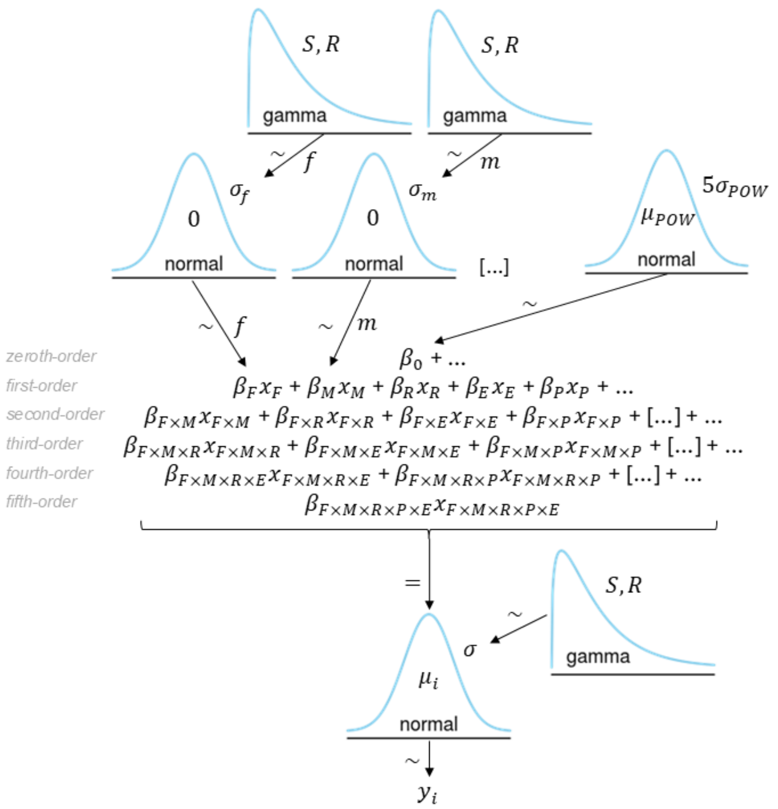

Figure S1.1. Bayesian model to fit the data for each epoch y_i. Letters represent the predictor factors for cue frequency ($\vec{x}_F$, with 2 levels f), cue modality ($\vec{x}_M$, with 2 levels m), cue rhythmicity ($\vec{x}_R$, with 2 levels r), EEG electrode ($\vec{x}_E$, with 30 levels e), and participants ($\vec{x}_P$, with 17 levels p). ~ denotes a stochastic relationship; = denotes a deterministic relation. Diagram following Kruschke (3) was generated using https://github.com/rasmusab/distribution_diagrams.

Markov Chain Monte Carlo (MCMC) sampling

The statistical model was fitted to the data using the Markov Chain Monte Carlo (MCMC) sampling technique, implemented in JAGS (4) with the MATLAB interface matJAGS (5). The MCMC sampler generated 50,000 samples for Model 1 and 100,000 samples for Model 2, using 3 chains. The first 5,000 samples and 10,000 samples were discarded for burn-in, for Model 1 and 2 respectively.

We ensured good sampling performance by evaluating the convergence of chains and the goodness of fit of the model. The convergence of chains, assessed by the Gelman-Rubin statistic ($\hat{R}$) (6) and the effective sample size (ESS) (3), for all parameters confirmed a good sampling performance (Table S1). $\hat{R}$ was 1.00 for all outcome measures, and the minimum ESS was 3008 for ITI-CV.

Table S1. Descriptive of the Markov Chain Monte Carlo (MCMC) sampling performance assessed by the effective sampling size (ESS) and Gelman-Rubin statistic ($\hat{R}$) for the outcome measures. Values represent the mean (standard deviation) and the [minimum and maximum] values of the ESS and $\hat{R}$.

	**Effective sample size (ESS)**		**Gelman-Rubin statistic ($\hat{R}$)**	
	Mean (SD)	[min – max]	Mean (SD)	[min – max]
Beta power before tap	49589 (2552)	[12665-50001]	1,0000 (0,0001)	[1,0000-1,0008]
Beta power after tap	49535 (2920)	[12272-50001]	1,0000 (0,0001)	[1,0000-1,0007]
ITI-D	90432(25886)	[6667-100002]	1,0000 (0,0001)	[1,0000-1,0002]
ITI-CV	73256 (34823)	[3008-100002]	1,0000 (0,0002)	[1,0000-1,0014]

Posterior Predictive Check

The goodness of fit of the model was examined by visual inspection of the posterior distributions and the measured data per condition, participant, and electrode (Figure S1.3). Moreover, the Pearson correlation coefficient between the measured and estimated data, using the average per level of each factor, further confirmed that the models fitted the data adequately. The lowest correlation coefficient of 0.976 was found for the ITI-CV.

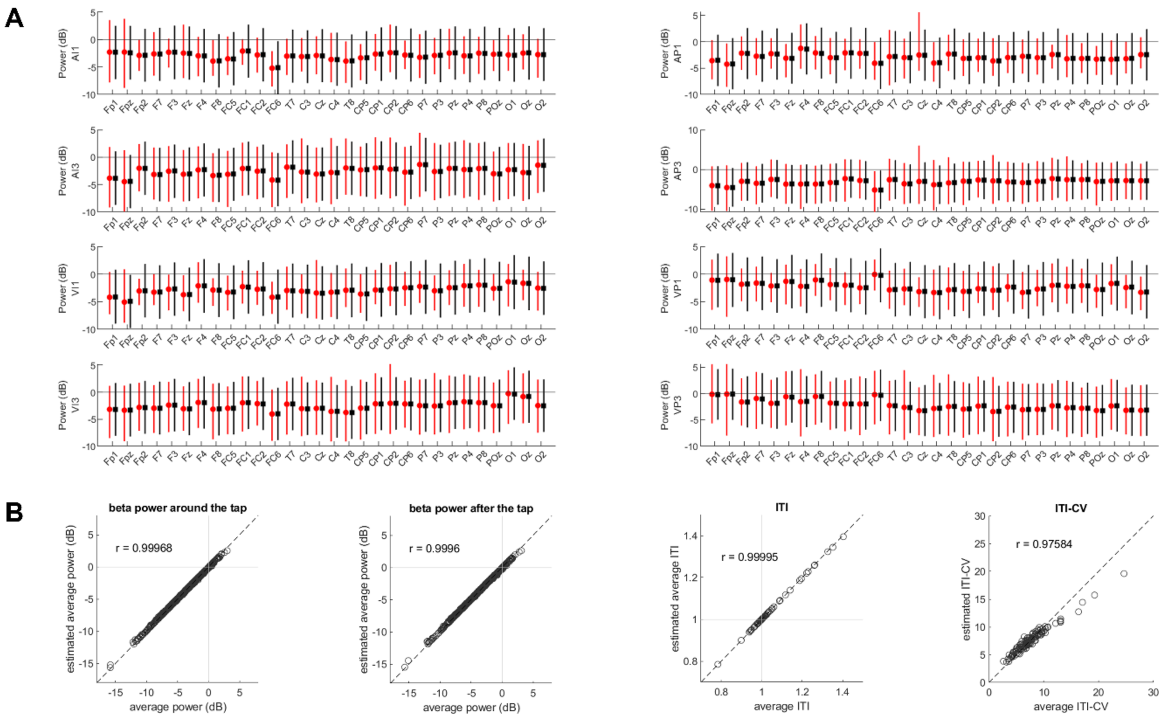

Figure S1.3. Evaluation of the goodness of fit of the hierarchical Bayesian model. **A:** Comparison of beta power (dB) of the measured EEG data (red circles) and the posterior distribution as estimated by the Bayesian model (black squares) for a single participant (sub 1). Mean and 95% highest density interval are shown for each electrode per cue condition (subplots). **B:** The average measured data vs. the average of the posterior distribution (model prediction) for, from left to right, the beta power around the tap, beta power after the tap, ITI-D, and ITI-CV. r = Pearson correlation coefficient.

Considerations calculation power

The power is calculated by squaring the amplitude of the signal, which results in a positively skewed distribution. Therefore, we determined the power of the beta band in decibels (dB). The decibel transformation that we applied to the data as baseline normalization, results in normally distributed power and allows the use of normally distributed priors in Bayesian models. In contrast, the percentage change in power, known as event-related (de)synchronization and commonly used in literature (7), remains positively skewed. Skewed prior distributions, such as the gamma distribution, cannot deal with both positive and negative values. Hence, the percentage change in power is unsuitable for Bayesian statistical analyses, making the decibel transformation the preferred choice.

Another consideration involved applying the decibel transformation to epoch data rather than averaged data, as typically advised to reduce sensitivity to outliers (8). However, Bayesian models require data from all trials to accurately model and achieve chain convergence. As a consequence, the decibel transformation emphasizes negative values, for which we adjusted by subtracting the participant’s average beta power across all data of all electrodes.

References

1. Kruschke JK. Bayesian estimation supersedes the t test. J Exp Psychol Gen. 2013;142(2):573-603.
2. Wagenmakers EJ, Marsman M, Jamil T, Ly A, Verhagen J, Love J, et al. Bayesian inference for psychology. Part I: Theoretical advantages and practical ramifications. Psychonomic bulletin & review. 2018;25(1):35-57.
3. Kruschke JK. Doing Bayesian Data Analysis: a Tutorial with R, JAGS, and Stan. Second Edition ed: Academic Press / Elsevier; 2015. p. 583-620.
4. Plummer M. JAGS: A Program for Analysis of Bayesian Graphical Models using Gibbs Sampling. 3rd International Workshop on Distributed Statistical Computing (DSC 2003); Vienna, Austria. 2003;124.
5. Carroll MSC. MATJAGS, a Matlab interface for JAGS Version 1.3.3. 2016 [Available from: https://github.com/msteyvers/matjags.
6. Gelman A, Rubin DB. Inference from Iterative Simulation Using Multiple Sequences. Statistical Science. 1992;7(4):457-72, 16.
7. Pfurtscheller G, Lopes da Silva FH. Event-related EEG/MEG synchronization and desynchronization: basic principles. Clinical neurophysiology : official journal of the International Federation of Clinical Neurophysiology. 1999;110(11):1842-57.
8. Cohen MX. Analyzing Neural Time Series Data: Theory and Practice. Issues in clinical and cognitive neuropsychology. Cambridge, Massachusetts: The MIT Press; 2014. p. 217-40.

S2. Supplementary results

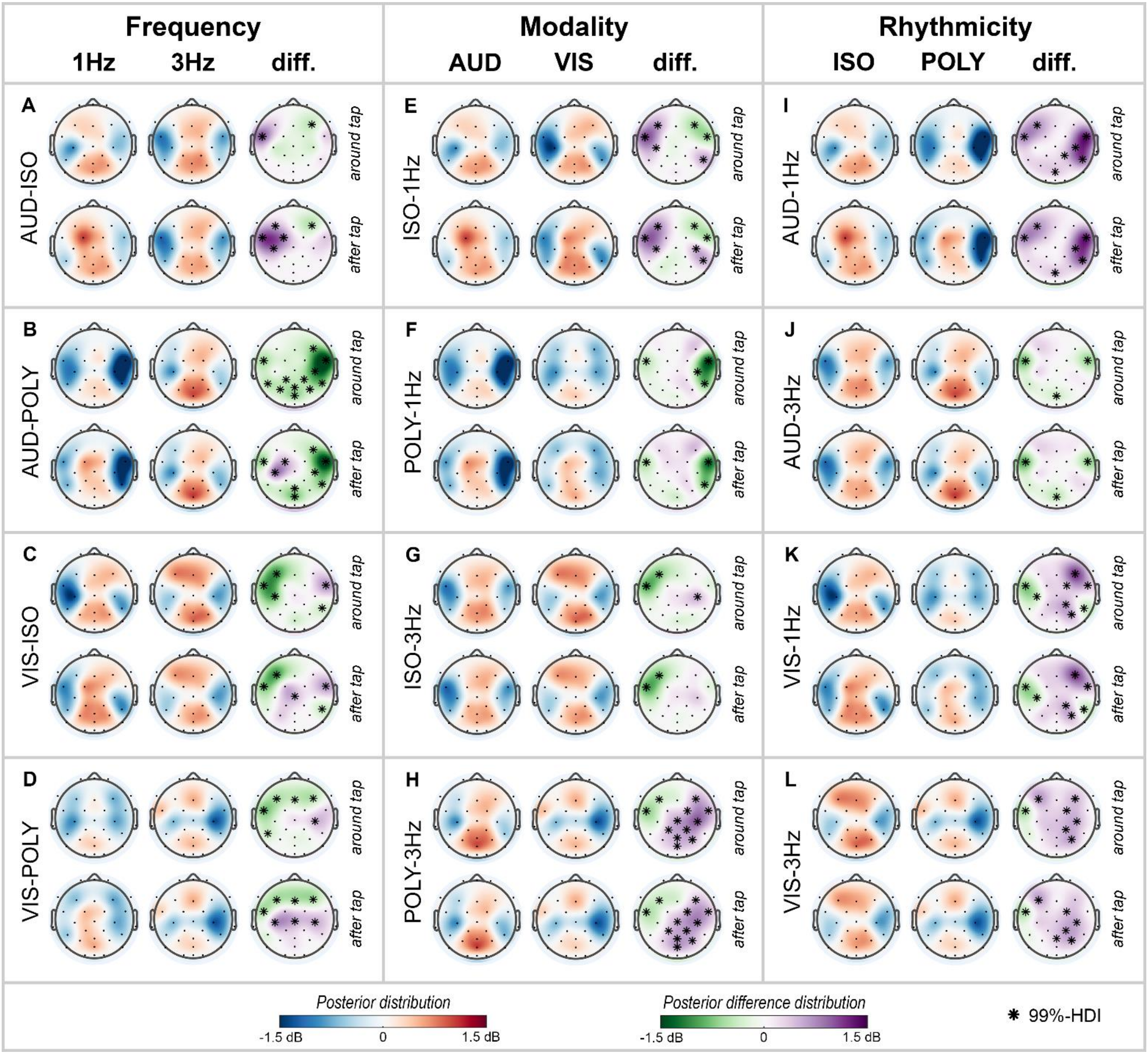

Figure S2.1. The posterior distribution (blue-red) of the beta band activity around the tap (top) and after the tap (bottom) and the posterior difference distributions (green-purple) of the beta band for all comparisons of interest, to evaluate the effect of cue frequency (1 Hz vs. 3 Hz; left, A-D), the effect of cue modality (auditory vs. visual; middle, E-H) and the effect of cue rhythmicity (isorhythmic vs. polyrhythmic; right, I-L). * Asterisks indicate electrode positions for which the 99%-HDI of the posterior difference distribution excludes 0 dB, and the average absolute posterior difference distribution is larger than 0.4 dB.

Table S2.1. Descriptives (mean and [99% highest density interval (99%-HDI)]) of the posterior difference distribution for comparisons of interest to evaluate the effect of cue frequency (1 Hz cues vs. 3 Hz cues), for all electrodes and both time windows (around the finger tap and after the finger tap). * Asterisks indicate electrode positions for which the 99%-HDI of the posterior difference distribution excludes 0 dB, and the average absolute posterior difference distribution is larger than 0.4 dB.

	**AUD-ISO (1Hz – 3Hz)**		**AUD-POLY (1Hz – 3Hz)**		**VIS-ISO (1Hz – 3Hz)**		**VIS-POLY (1Hz – 3Hz)**	
	around tap	*after tap*	*around tap*	*after tap*	*around tap*	*after tap*	*around tap*	*after tap*
	**mean 99%-HDI**	**mean 99%-HDI**	**mean 99%-HDI**	**mean 99%-HDI**	**mean 99%-HDI**	**mean 99%-HDI**	**mean 99%-HDI**	**mean 99%-HDI**
F3	0,33 [0,05 0,61]	0,52 [0,23 0,81]*	-0,10 [-0,48 0,28]	0,05 [-0,34 0,44]	-1,13 [-1,41 -0,84]*	-1,09 [-1,39 -0,79]*	-0,60 [-0,99 -0,21]*	-0,62 [-1,01 -0,22]*
Fz	-0,17 [-0,45 0,11]	-0,04 [-0,33 0,25]	-0,28 [-0,66 0,10]	-0,13 [-0,52 0,26]	-0,38 [-0,67 -0,10]	-0,21 [-0,51 0,08]	-0,43 [-0,82 -0,04]*	-0,52 [-0,91 -0,13]*
F4	-0,45 [-0,74 -0,17]*	-0,51 [-0,81 -0,22]*	-0,75 [-1,13 -0,37]*	-0,62 [-1,01 -0,23]*	0,20 [-0,09 0,48]	0,17 [-0,13 0,47]	-0,53 [-0,91 -0,14]*	-0,61 [-1,01 -0,21]*
FC5	1,07 [0,79 1,36]*	1,14 [0,85 1,43]*	-0,63 [-1,02 -0,25]*	-0,66 [-1,05 -0,27]*	-1,01 [-1,29 -0,72]*	-0,80 [-1,10 -0,50]*	-0,81 [-1,20 -0,42]*	-0,63 [-1,03 -0,24]*
FC1	-0,11 [-0,39 0,16]	0,83 [0,54 1,13]*	-0,31 [-0,69 0,06]	0,54 [0,15 0,93]*	-0,13 [-0,41 0,15]	0,39 [0,09 0,69]	-0,09 [-0,47 0,30]	0,40 [0,00 0,79]
FC2	-0,24 [-0,51 0,04]	-0,05 [-0,35 0,24]	-0,40 [-0,78 -0,01]	-0,16 [-0,55 0,23]	-0,13 [-0,42 0,15]	0,14 [-0,16 0,44]	-0,11 [-0,49 0,28]	0,22 [-0,18 0,61]
FC6	0,30 [0,02 0,58]	0,34 [0,04 0,64]	-1,79 [-2,17 -1,41]*	-1,72 [-2,11 -1,33]*	0,74 [0,46 1,02]*	0,72 [0,42 1,02]*	0,34 [-0,05 0,73]	0,19 [-0,21 0,58]
C3	-0,25 [-0,53 0,03]	0,91 [0,61 1,21]*	-0,06 [-0,44 0,32]	0,94 [0,56 1,33]*	-0,88 [-1,16 -0,60]*	-0,07 [-0,37 0,23]	-0,24 [-0,62 0,15]	0,71 [0,32 1,10]*
Cz	-0,17 [-0,45 0,11]	0,25 [-0,04 0,55]	-0,30 [-0,68 0,08]	0,20 [-0,20 0,59]	0,16 [-0,13 0,44]	0,53 [0,23 0,84]*	0,22 [-0,17 0,61]	0,60 [0,21 1,00]*
C4	-0,32 [-0,60 -0,04]	0,21 [-0,08 0,51]	-1,26 [-1,64 -0,87]*	-0,62 [-1,02 -0,23]*	0,09 [-0,20 0,37]	0,06 [-0,24 0,35]	0,49 [0,11 0,88]*	0,56 [0,16 0,97]*
CP5	0,06 [-0,22 0,33]	0,20 [-0,09 0,49]	-0,55 [-0,93 -0,17]*	-0,33 [-0,73 0,06]	-0,34 [-0,62 -0,05]	-0,18 [-0,47 0,12]	-0,40 [-0,79 -0,01]*	-0,22 [-0,62 0,17]
CP1	-0,24 [-0,52 0,03]	0,08 [-0,22 0,37]	-0,46 [-0,84 -0,08]*	-0,09 [-0,48 0,30]	-0,06 [-0,34 0,22]	0,35 [0,05 0,65]	-0,02 [-0,42 0,36]	0,38 [-0,01 0,77]
CP2	-0,16 [-0,44 0,12]	0,00 [-0,30 0,29]	-0,55 [-0,93 -0,18]*	-0,25 [-0,64 0,14]	0,04 [-0,24 0,33]	0,08 [-0,22 0,38]	-0,04 [-0,43 0,34]	0,14 [-0,26 0,54]
CP6	0,18 [-0,10 0,45]	0,09 [-0,21 0,38]	-0,80 [-1,18 -0,42]*	-0,70 [-1,09 -0,31]*	-0,42 [-0,70 -0,13]*	-0,47 [-0,76 -0,17]*	0,20 [-0,18 0,59]	0,26 [-0,14 0,66]
P3	0,04 [-0,24 0,32]	0,06 [-0,24 0,35]	-0,47 [-0,85 -0,09]*	-0,39 [-0,78 0,01]	0,03 [-0,25 0,31]	0,35 [0,05 0,65]	-0,06 [-0,45 0,32]	0,08 [-0,32 0,47]
Pz	-0,11 [-0,39 0,17]	-0,01 [-0,32 0,28]	-0,55 [-0,93 -0,17]*	-0,48 [-0,87 -0,08]*	-0,16 [-0,44 0,13]	0,06 [-0,24 0,36]	-0,16 [-0,54 0,22]	0,21 [-0,19 0,61]
P4	0,00 [-0,28 0,27]	0,03 [-0,27 0,32]	-0,43 [-0,80 -0,05]*	-0,33 [-0,72 0,06]	-0,16 [-0,44 0,12]	0,03 [-0,26 0,33]	-0,09 [-0,48 0,30]	0,03 [-0,36 0,43]
POz	0,08 [-0,20 0,36]	0,09 [-0,20 0,38]	-0,75 [-1,13 -0,37]*	-0,87 [-1,26 -0,47]*	-0,37 [-0,65 -0,08]	0,04 [-0,26 0,33]	-0,10 [-0,48 0,28]	0,19 [-0,20 0,59]

Table S2.2. Descriptives (mean and [99% highest density interval (99%-HDI)]) of the posterior difference distribution for comparisons of interest to evaluate the effect of cue modality (auditory cues vs. visual cues), for all electrodes and both time windows (around the finger tap and after the finger tap). * Asterisks indicate electrode positions for which the 99%-HDI of the posterior difference distribution excludes 0 dB, and the average absolute posterior difference distribution is larger than 0.4 dB.

	**ISO-1Hz (AUD – VIS)**		**POLY-1Hz (AUD – VIS)**		**ISO-3Hz (AUD – VIS)**		**POLY-3Hz (AUD – VIS)**	
	around tap	*after tap*	*around tap*	*after tap*	*around tap*	*after tap*	*around tap*	*after tap*
	**mean 99%-HDI**	**mean 99%-HDI**	**mean 99%-HDI**	**mean 99%-HDI**	**mean 99%-HDI**	**mean 99%-HDI**	**mean 99%-HDI**	**mean 99%-HDI**
F3	0,66 [0,32 1,00]*	0,78 [0,43 1,13]*	0,09 [-0,40 0,58]	0,25 [-0,25 0,75]	-0,80 [-1,01 -0,59]*	-0,83 [-1,05 -0,60]*	-0,41 [-0,63 -0,19]*	-0,42 [-0,65 -0,18]*
Fz	-0,08 [-0,41 0,26]	0,01 [-0,34 0,37]	-0,05 [-0,54 0,44]	0,11 [-0,39 0,62]	-0,29 [-0,50 -0,08]	-0,16 [-0,38 0,07]	-0,20 [-0,42 0,02]	-0,28 [-0,51 -0,05]
F4	-0,64 [-0,98 -0,30]*	-0,53 [-0,89 -0,18]*	0,35 [-0,14 0,84]	0,43 [-0,08 0,93]	0,01 [-0,21 0,22]	0,15 [-0,07 0,37]	0,57 [0,34 0,79]*	0,43 [0,19 0,66]*
FC5	1,10 [0,76 1,44]*	0,95 [0,60 1,31]*	-0,51 [-1,00 -0,02]*	-0,57 [-1,07 -0,06]*	-0,98 [-1,19 -0,77]*	-0,98 [-1,21 -0,76]*	-0,68 [-0,90 -0,47]*	-0,53 [-0,76 -0,30]*
FC1	0,00 [-0,33 0,33]	0,39 [0,04 0,74]	-0,04 [-0,53 0,45]	0,23 [-0,27 0,73]	-0,02 [-0,23 0,20]	-0,05 [-0,28 0,17]	0,19 [-0,03 0,41]	0,09 [-0,15 0,31]
FC2	-0,14 [-0,48 0,20]	-0,07 [-0,42 0,28]	0,14 [-0,36 0,64]	0,16 [-0,35 0,66]	-0,04 [-0,25 0,17]	0,12 [-0,10 0,35]	0,43 [0,22 0,65]*	0,53 [0,30 0,76]*
FC6	-0,66 [-1,00 -0,33]*	-0,60 [-0,95 -0,24]*	-1,50 [-2,00 -1,01]*	-1,35 [-1,85 -0,85]*	-0,22 [-0,43 -0,01]	-0,22 [-0,44 0,00]	0,62 [0,40 0,84]*	0,55 [0,32 0,78]*
C3	0,42 [0,09 0,75]*	0,63 [0,27 0,98]*	-0,08 [-0,57 0,41]	-0,02 [-0,52 0,47]	-0,20 [-0,41 0,01]	-0,36 [-0,58 -0,13]	-0,25 [-0,47 -0,03]	-0,25 [-0,48 -0,02]
Cz	-0,15 [-0,48 0,19]	-0,09 [-0,45 0,27]	0,10 [-0,40 0,59]	0,14 [-0,37 0,65]	0,18 [-0,03 0,40]	0,18 [-0,04 0,41]	0,62 [0,40 0,84]*	0,54 [0,30 0,77]*
C4	0,12 [-0,22 0,46]	0,52 [0,17 0,88]*	-0,73 [-1,22 -0,24]*	-0,39 [-0,89 0,12]	0,53 [0,31 0,74]*	0,37 [0,14 0,59]	1,02 [0,80 1,24]*	0,80 [0,56 1,03]*
CP5	0,27 [-0,06 0,61]	0,19 [-0,16 0,54]	-0,17 [-0,67 0,33]	-0,17 [-0,68 0,34]	-0,12 [-0,33 0,09]	-0,19 [-0,41 0,03]	-0,03 [-0,25 0,19]	-0,06 [-0,29 0,18]
CP1	-0,11 [-0,44 0,23]	-0,16 [-0,52 0,19]	0,06 [-0,44 0,55]	-0,06 [-0,56 0,45]	0,08 [-0,14 0,29]	0,11 [-0,11 0,33]	0,49 [0,27 0,71]*	0,41 [0,18 0,64]*
CP2	0,01 [-0,32 0,35]	-0,02 [-0,38 0,33]	0,20 [-0,29 0,70]	0,26 [-0,24 0,76]	0,22 [0,00 0,43]	0,07 [-0,16 0,29]	0,71 [0,49 0,93]*	0,65 [0,41 0,88]*
CP6	0,65 [0,31 0,98]*	0,61 [0,25 0,96]*	-0,90 [-1,39 -0,41]*	-0,84 [-1,34 -0,34]*	0,06 [-0,15 0,27]	0,05 [-0,17 0,28]	0,10 [-0,12 0,32]	0,12 [-0,12 0,35]
P3	-0,01 [-0,35 0,32]	-0,25 [-0,61 0,10]	-0,13 [-0,62 0,36]	-0,17 [-0,67 0,34]	-0,02 [-0,23 0,19]	0,04 [-0,18 0,27]	0,28 [0,06 0,51]	0,30 [0,07 0,53]
Pz	0,01 [-0,32 0,35]	-0,14 [-0,50 0,21]	0,09 [-0,40 0,59]	-0,07 [-0,58 0,44]	-0,04 [-0,25 0,18]	-0,07 [-0,29 0,16]	0,49 [0,27 0,71]*	0,62 [0,39 0,85]*
P4	-0,02 [-0,35 0,32]	0,00 [-0,35 0,35]	0,20 [-0,29 0,69]	0,18 [-0,32 0,68]	-0,17 [-0,38 0,04]	0,00 [-0,22 0,23]	0,53 [0,31 0,75]*	0,54 [0,32 0,77]*
POz	0,16 [-0,18 0,49]	-0,03 [-0,38 0,33]	-0,18 [-0,68 0,31]	-0,39 [-0,90 0,11]	-0,29 [-0,50 -0,07]	-0,09 [-0,31 0,14]	0,47 [0,25 0,69]*	0,67 [0,44 0,90]*

Table S2.3. Descriptives (mean and [99% highest density interval (99%-HDI)]) of the posterior difference distribution for comparisons of interest to evaluate the effect of rhythmicity (isorhythmic cues vs. polyrhythmic cues), for all electrodes and both time windows (around the finger tap and after the finger tap). * Asterisks indicate electrode positions for which the 99%-HDI of the posterior difference distribution excludes 0 dB, and the average absolute posterior difference distribution is larger than 0.4 dB.

	**AUD-1Hz (ISO – POLY)**		**AUD-3Hz (ISO – POLY)**		**VIS-1Hz (ISO – POLY)**		**VIS-3Hz (ISO – POLY)**	
	around tap	*after tap*	*around tap*	*after tap*	*around tap*	*after tap*	*around tap*	*after tap*
	**mean 99%-HDI**	**mean 99%-HDI**	**mean 99%-HDI**	**mean 99%-HDI**	**mean 99%-HDI**	**mean 99%-HDI**	**mean 99%-HDI**	**mean 99%-HDI**
F3	0,72 [0,38 1,06]*	0,73 [0,38 1,08]*	0,29 [0,15 0,43]	0,26 [0,11 0,41]	0,16 [-0,18 0,50]	0,20 [-0,16 0,55]	0,68 [0,53 0,84]*	0,67 [0,50 0,83]*
Fz	0,21 [-0,12 0,56]	0,26 [-0,10 0,61]	0,10 [-0,04 0,24]	0,17 [0,02 0,32]	0,24 [-0,10 0,59]	0,36 [0,00 0,71]	0,19 [0,04 0,35]	0,05 [-0,11 0,22]
F4	0,23 [-0,11 0,57]	0,21 [-0,14 0,57]	-0,07 [-0,21 0,07]	0,10 [-0,04 0,26]	1,22 [0,88 1,56]*	1,17 [0,82 1,52]*	0,49 [0,34 0,65]*	0,38 [0,22 0,55]
FC5	0,84 [0,50 1,18]*	0,82 [0,47 1,17]*	-0,87 [-1,01 -0,73]*	-0,98 [-1,13 -0,83]*	-0,77 [-1,11 -0,43]*	-0,70 [-1,05 -0,34]*	-0,57 [-0,73 -0,42]*	-0,53 [-0,69 -0,37]*
FC1	0,31 [-0,03 0,66]	0,38 [0,03 0,73]	0,11 [-0,03 0,25]	0,09 [-0,06 0,24]	0,27 [-0,07 0,62]	0,22 [-0,13 0,57]	0,32 [0,16 0,47]	0,23 [0,06 0,39]
FC2	0,12 [-0,22 0,46]	0,11 [-0,24 0,46]	-0,04 [-0,18 0,10]	0,00 [-0,15 0,15]	0,40 [0,06 0,75]*	0,33 [-0,02 0,68]	0,43 [0,27 0,58]*	0,41 [0,25 0,58]*
FC6	1,36 [1,02 1,70]*	1,32 [0,97 1,67]*	-0,72 [-0,86 -0,58]*	-0,74 [-0,89 -0,59]*	0,52 [0,18 0,87]*	0,57 [0,22 0,92]*	0,12 [-0,03 0,27]	0,04 [-0,12 0,20]
C3	0,06 [-0,28 0,40]	0,15 [-0,20 0,50]	0,25 [0,10 0,39]	0,18 [0,03 0,33]	-0,44 [-0,78 -0,10]*	-0,50 [-0,86 -0,16]*	0,20 [0,04 0,35]	0,28 [0,12 0,44]
Cz	0,09 [-0,25 0,43]	0,05 [-0,30 0,40]	-0,04 [-0,18 0,10]	-0,01 [-0,16 0,14]	0,33 [-0,02 0,67]	0,28 [-0,07 0,64]	0,39 [0,24 0,55]	0,35 [0,18 0,51]
C4	1,01 [0,67 1,35]*	0,96 [0,60 1,31]*	0,07 [-0,07 0,22]	0,12 [-0,03 0,27]	0,16 [-0,18 0,50]	0,05 [-0,31 0,40]	0,57 [0,41 0,72]*	0,56 [0,39 0,72]*
CP5	0,27 [-0,07 0,61]	0,25 [-0,10 0,60]	-0,33 [-0,47 -0,19]	-0,29 [-0,44 -0,14]	-0,17 [-0,51 0,18]	-0,11 [-0,46 0,24]	-0,23 [-0,39 -0,08]	-0,16 [-0,32 0,01]
CP1	0,18 [-0,17 0,52]	0,13 [-0,22 0,48]	-0,04 [-0,18 0,10]	-0,04 [-0,19 0,11]	0,34 [0,00 0,68]	0,23 [-0,11 0,58]	0,38 [0,22 0,53]	0,26 [0,10 0,42]
CP2	0,42 [0,09 0,76]*	0,27 [-0,08 0,63]	0,03 [-0,11 0,17]	0,03 [-0,12 0,18]	0,61 [0,27 0,96]*	0,55 [0,20 0,90]*	0,52 [0,37 0,68]*	0,61 [0,44 0,77]*
CP6	1,09 [0,75 1,44]*	1,02 [0,67 1,37]*	0,12 [-0,02 0,26]	0,23 [0,08 0,38]	-0,45 [-0,79 -0,11]*	-0,43 [-0,78 -0,07]*	0,16 [0,01 0,32]	0,30 [0,14 0,47]
P3	0,34 [-0,01 0,68]	0,24 [-0,12 0,59]	-0,17 [-0,32 -0,03]	-0,21 [-0,36 -0,06]	0,22 [-0,12 0,56]	0,32 [-0,03 0,68]	0,14 [-0,02 0,29]	0,05 [-0,11 0,22]
Pz	0,29 [-0,05 0,64]	0,18 [-0,18 0,53]	-0,15 [-0,29 -0,01]	-0,29 [-0,43 -0,14]	0,37 [0,03 0,72]	0,25 [-0,10 0,60]	0,38 [0,22 0,53]	0,40 [0,24 0,57]*
P4	0,35 [0,01 0,69]	0,38 [0,03 0,73]	-0,07 [-0,21 0,07]	0,02 [-0,13 0,17]	0,57 [0,22 0,91]*	0,56 [0,21 0,91]*	0,64 [0,48 0,79]*	0,56 [0,40 0,72]*
POz	0,41 [0,06 0,75]*	0,44 [0,08 0,79]*	-0,42 [-0,57 -0,28]*	-0,52 [-0,67 -0,37]*	0,07 [-0,27 0,42]	0,08 [-0,28 0,43]	0,33 [0,18 0,49]	0,24 [0,08 0,40]
